# Supplementary material for: Sexual risk behaviour, sexual victimisation, substance use and other factors related to depression in men who have sex with men in Wenzhou, China: a cross-sectional study
Source: BMJ Open. 2018 May 3;8(4):bmjopen-2016-013512. doi: 10.1136/bmjopen-2016-013512 (PMC5969728; doi:10.1136/bmjopen-2016-013512)
Supplement: Author's manuscript [file bmjopen-2016-013512.draft_revisions.pdf]

BMJ Open

BMJ Open is committed to open peer review. As part of this commitment we make the peer review history of every article we publish publicly available.

When an article is published we post the peer reviewers' comments and the authors' responses online. We also post the versions of the paper that were used during peer review. These are the versions that the peer review comments apply to.

The versions of the paper that follow are the versions that were submitted during the peer review process. They are not the versions of record or the final published versions. They should not be cited or distributed as the published version of this manuscript.

BMJ Open is an open access journal and the full, final, typeset and author-corrected version of record of the manuscript is available on our site with no access controls, subscription charges or pay-per-view fees (http://bmjopen.bmj.com).

If you have any questions on BMJ Open's open peer review process please email info.bmjopen@bmj.com

Sexual risk behavior, sexual victimization, substance use, and other factors related to depression in men who have sex with men in Wenzhou, China: a cross-sectional study

Journal:	*BMJ Open*
Manuscript ID	bmjopen-2016-013512
Article Type:	Research
Date Submitted by the Author:	08-Aug-2016
Complete List of Authors:	Pan, Xiaohong; Center for Disease Control and Prevention of Zhejiang Province<br>Li, Runhua; Ningbo University, Medical school<br>Ma, Qiaoqin; Center for Disease Control and Prevention of Zhejiang Province, ;<br>Wang, Hui; Center for Disease Control and Prevention of Zhejiang Province,<br>Jiang, Tingting; Center for Disease Control and Prevention of Zhejiang Province,<br>He, Lin; Center for Disease Control and Prevention of Zhejiang Province,<br>Zeng, Shidian; Wenzhou Center for Disease Control and Prevention,<br>Wang, Dayong; Wenzhou Center for Disease Control and Prevention,<br>Ye, Zhenmiao; Wenzhou Center for Disease Control and Prevention,<br>Zhu, Haishen; Center for Disease Control and Prevention of Lucheng District, Wenzhou<br>Zhao, Dongshe; Lucheng Center for Disease Control and Prevention<br>Xia, Shichang; Center for Disease Control and Prevention of Zhejiang Province,
**Primary Subject Heading**:	Public health
Secondary Subject Heading:	HIV/AIDS, Mental health
Keywords:	PUBLIC HEALTH, HIV & AIDS < INFECTIOUS DISEASES, MENTAL HEALTH

Sexual risk behavior, sexual victimization, substance use, and other factors related to depression in men who have sex with men in Wenzhou, China: a cross-sectional study

#Xiaohong Pan[1], #Runhua Li[1,2], Qiaoqin Ma[1], Hui Wang[1], Tingting Jiang[1], Lin He[1], Shidian Zeng[3], Dayong Wang[3], Zhenmiao Ye[3], Haishen Zhu[4], Dongshe Zhao[4], *Shichang Xia[1]

[1]Center for Disease Control and Prevention of Zhejiang Province, Hangzhou, Zhejiang, China

[2]Department of Epidemiology & Biostatistics, School of Medicine, Ningbo University, Ningbo, Zhejiang, China

[3]Wenzhou Center for Disease Control and Prevention, Wenzhou, Zhejiang, China

[4] Lucheng Center for Disease Control and Prevention, Wenzhou, Zhejiang, China.

*Corresponding author: Shichang Xia

Email: shchxia@cdc.zj.cn

#Xiaohong Pan, and Runhua Li contributed equally to this work.

For peer review only - http://bmjopen.bmj.com/site/about/guidelines.xhtml

Abstract

Objectives: Men who have sex with men (MSM) are a marginalized population at high risk for a variety of mental health problems that may mutually facilitate HIV transmission. We assessed factors associated with depressive symptoms among MSM, which can provide some guidance for future mental health interventions with the MSM population for prevention of HIV infection and transmission. **Methods:** A cross-sectional study was conducted in Wenzhou City, China using respondent-driven sampling between December 2013 and June 2014. A face-to-face questionnaire survey was employed to collect information about mental and psychosocial health conditions and sexual behavior characteristics among MSM. Bivariate and multivariate logistic regression analyses were used to identify factors associated with depression. **Results:** Of the 454 participants in the study, prevalence of major depression was 34.6%. During the past 6 months, 85% had anal sex, rate of consistent condom use during anal intercourse was 45.1%, 76.7% had oral sex, and percentage of consistent condom use during oral sex was 19.8%. Of the respondents, 16.1% reported suicidal ideation, 62.6% had a history of smoking, and 41.4% had a history of drinking alcohol, of which 46.3% reported that they were once drunk. Drug abuse was reported in 3.7% of the sample. Adult sexual violence from same-sex partners was 16.1%, and 5.1% reported childhood sexual abuse. A multivariate analysis showed that depression was associated with younger age; no consistent condom use during anal sex in the past 6 months; suicidal ideation; adult sexual violence from male partners; self-perceived possibility of HIV infection; and not knowing about

For peer review only - http://bmjopen.bmj.com/site/about/guidelines.xhtml

antiretroviral therapy for HIV/AIDS. **Conclusion:** We found high levels of depression, unprotected sex, and suicidal ideation among MSM. Comprehensive intervention strategies that combine psychological, behavioral, and social aspects are needed to address the mental health issues of MSM, with special attention given to suicidality and high-risk behaviors.

Strengths and limitations of this study

1. The present study obtained the prevalence of major depression, sexual risk behavior, sexual victimization, suicidality, substance use and elucidated some related syndemic factors to depression among MSM in Wenzhou with high HIV prevalence.

2. Our study can provide some guidance for mental health and HIV prevention interventions for the MSM population.

3. It is unavoidable that MSM might underreport some sensitive issues such as unprotected sex, drug abuse, and sexual violence covered in the questionnaire in the rather homophobic and hostile environment of China.

4. The causality of depression and associated risk factors is not inferred due to the cross-sectional design, and the relevant information is subjected to recall bias, which may have an influence on the magnitude of associations with depression.

For peer review only

For peer review only - http://bmjopen.bmj.com/site/about/guidelines.xhtml

Introduction

Men who have sex with men (MSM) are a marginalized population facing many challenges including stigma, abuse, social and legal discrimination, and lack of social support,[1-3] which may result in high risk for a variety of mental and psychosocial health problems, such as depression, suicidality, and drug and alcohol abuse.[4-7] A large prospective cohort study showed that syndemics of depressive symptoms, heavy alcohol use and drug use, and childhood sexual abuse (CSA) increased HIV risk and HIV-related risk behaviors.[8] Depression and substance abuse among MSM have been linked to unprotected anal intercourse (UAI) and group sex,[9,10] which was the predominant factor of HIV acquisition and transmission.[11,12] These interactive factors have resulted in high HIV prevalence and incidence among MSM. It is estimated that MSM accounted for 29.4 % of the new HIV infections in China in 2011.[13] In Wenzhou City of China, the prevalence of HIV was up to 22.8% in a previous publication.[14]

A survey conducted in four large American cities showed the prevalence of depressive symptoms among MSM was 29.2%, nearly three times that of the general population.[15] In China, the prevalence of depressive symptoms among MSM varied from 34.8% to 63.9%,[16-20] which was much higher than 5.3% among the general population.[21] Previous studies showed that factors associated with depression among MSM included race,[22] lower educational level,[23] younger age,[24] and marriage to a woman.[25,26] Psychological factors such as sexual victimization,[27,28] substance abuse,[29,30] and HIV or sexual stigma[25] were associated with depressive symptoms among MSM. Some studies

For peer review only

For peer review only - http://bmjopen.bmj.com/site/about/guidelines.xhtml

showed that engaging in high-risk sexual behaviors was associated with depressive symptoms among the MSM population.[31,32] Few studies have examined the associations between HIV-related cognitions, such as the extent of knowing about antiretroviral therapy (ART) for HIV-infected individuals and history of taking the HIV test, and depression, which is very important to increase HIV/AIDS knowledge and eliminate phobia to HIV/AIDS. These previous studies were conducted to examine several independent factors associated with depression and did not comprehensively consider integrating sociodemographic characteristics, high-risk sexual behaviors, psychosocial factors, HIV-related cognitions, and HIV testing history into their analyses.

Some studies conducted in the US and in European countries examined depression and associated factors among MSM, while such research in China has been limited and lacks comprehensiveness. In the present study, our endeavor was to ascertain the prevalence of depression in Wenzhou City, China and related syndemic factors, including suicidality, sexual victimization, sexual risk behaviors, and HIV-related cognitions. Such knowledge would provide guidance for mental health and HIV prevention interventions for the MSM population.

Materials and methods

Study population and sampling

This cross-sectional study was conducted in Wenzhou City, China between December 2013 and June 2014. This baseline survey research was conducted for the

For peer review only

For peer review only - http://bmjopen.bmj.com/site/about/guidelines.xhtml

purposes of controlling HIV/AIDS new infections among the MSM population in Zhejiang Province, China. The study procedures and sampling strategies are described in detail elsewhere.[14] Briefly, participants were recruited using a respondent−driving sampling (RDS) method. Participants who were at least 14 years old, had resided in Wenzhou at least 3 months, self-reported having had anal sex and/or oral sex in the past 12 months, and were willing to participate in the survey met inclusion criteria in this study. First, we selected 5 initial active “seed” participants meeting inclusion criteria from the local MSM population. After completing the field investigation and relevant testing, “seed” participants were provided with 3 recruitment coupons, and through the incentive measures the “seed” participants then referred a maximum of 3 respondents from their social network to participate in the survey. These respondents continued to refer a maximum of 3 peers to the study and continued to recruit MSM participants until equilibrium was reached. The sample composition reached equilibrium when the characteristics of the sample were stable, irrelevant to the initial seeds by a number of recruitment waves, and would not change much with further recruitment of participants. All participants received remuneration in the amount of RMB 100 (approximately US$ 16) for participation and another RMB 100 as an incentive to refer peers to participate in the study. Finally, 5 seeds recruited 30, 61, 81, 103, and 174 MSM, respectively, with a total sample of 454 MSM by 14 waves of recruitment. All eligible participants were interviewed by trained staff from the Center for Disease Control and Prevention of Lucheng District, Wenzhou, who also collected the questionnaire about

For peer review only - http://bmjopen.bmj.com/site/about/guidelines.xhtml

sociodemographics, sexual risk behaviors, psychosocial conditions, HIV-related cognitions, and HIV testing history of participants.

Measures

Mental and psychosocial health conditions

Depression. Depressive symptoms were assessed using the Center for Epidemiological Studies Depression Scale (CES-D). The CES-D consists of 20 items designed to assess the experience of depression. Respondents indicate the extent to which each item statement describes their experience during the past week. Response options include the following: “rarely or none of the time (less than 1 day),” “some or a little of the time (1–2 days),” “occasionally or a moderate amount of the time (3–4 days),” and “most or all of the time (5–7 days).” Responses are on a 4-point Likert sale and scored 0–3. Level of depression was categorized as follows: certain depressive symptoms (CES-D score of 20 or higher), possible depressive symptoms (CES-D score of 16–19), and no depressive symptoms (CES-D score of 15 or lower). Thus, higher scores indicate higher levels of depression. A total scale score of 20 or higher was used to define major depression in our study.

Use of substances. Substance use included smoking cigarettes, alcohol consumption, and use of drugs. Cigarette smoking refers to smoking one or more cigarettes every day for more than a year or smoking more than 300 cigarettes in 3 months or less. Alcohol consumption refers to ethanol intake of 100 grams per week such as 250 grams of 40% volume of wine consumption or 1 kilogram of 10% volume of red wine intake. According

For peer review only

For peer review only - http://bmjopen.bmj.com/site/about/guidelines.xhtml

to the definition, participants were asked whether they had a history of smoking and drinking alcohol, whether they had been drunk in the past year, and whether they wanted or needed to reduce drinking in the past year. Drug abuse was defined as having an excessive amount of drug use in the past year, and included psychotropic drugs (e.g., sedative hypnotics), caffeine, wheat carlin, LSD, and narcotics (e.g., opiates, cocaine, cannabinoids, etc.).

Sexual victimization. Sexual victimization included childhood sexual abuse and adult sexual violence from same-sex partners. The two survey items asked “Have you suffered sexual abuse in your childhood?” and “Have you suffered adult sexual violence from a male sexual partner?”

Suicidal ideation. Suicidal ideation was assessed for over one’s lifetime. Participants were asked, “Have you ever had suicidal thoughts in the past?”

Sexual risk behaviors

Sexual risk behaviors included 6 items: 1) number of anal intercourse male partners in the past 6 months, 2) consistent condom use during anal sex with men, 3) number of oral sex male partners in the past 6 months, 4) consistent condom use during oral sex with men, 5) number of female sexual partners, and 6) consistent condom use during sex with women.

HIV-related cognitions and testing history

HIV-related cognitions included 6 items: 1) influence of social perception on sexual behaviors of MSM population, 2) self-perceived possibility of HIV infection, 3) estimate

For peer review only - http://bmjopen.bmj.com/site/about/guidelines.xhtml

of present HIV prevalence among MSM, 4) receiving AIDS/sexually transmitted diseases (STDs) propaganda intervention in the past year, 5) knowing about ART for HIV/AIDS, and 6) perceived necessity to accept early ART for HIV-positive people. HIV test history items asked whether the participant had HIV testing in his lifetime, in the past year, regular HIV testing in the past, and intends to take regular testing.

Statistical analysis

Data were entered into EpiData version 3.1 (http://www.epidata.dk/) via double entry. After data cleaning and verification, the data were processed using PASW Statistics 18.0 (SPSS Inc., Chicago, IL, USA) for statistical analysis. For descriptive analyses, the mean, standard deviation (SD), median, and interquartile range (IQR) were computed for continuous variables and frequencies for categorical variables. Multicollinearity was diagnosed by examining the variance inflation factors (VIF). Variables significantly associated with the outcome variable at the level of $P < .10$ in the univariate logistic analyses were included in the multivariate logistic regression model, calculating adjusted odds ratios (AOR) and 95% confidence intervals (CI). Statistical significance was defined as $P < .05$.

Ethics statement

The study was approved by the ethical review board of Zhejiang Provincial Center for Disease Control and Prevention. No harm was given to the participants by the study and we protected confidentiality of the participants. A written informed consent was signed by all participants during the survey.

For peer review only - http://bmjopen.bmj.com/site/about/guidelines.xhtml

Results

Sample characteristics

A total of 454 MSM participated in the study, and the characteristics of the sample are provided in Table 1. Of the 454 participants, 55.7% (253/454) were never married and 51.4% (233/453) resided in Wenzhou City for more than three years. The age of the respondents ranged from 17 to 76 years (median = 33; IQR, 26–41). Regarding educational level, 86.6% (393/454) had an education of senior school or lower. Roughly sixty percent (57.3%) of the participants had an income of not less than 3000 RMB monthly, 30.8% (140/454) had health insurance, and 40.5% reported their sexual orientation as homosexual or gay.

Depression, sexual risk behaviors, suicidality, substance use, sexual violence, HIV-related cognitions, and HIV testing history

The median CES-D scale score was 16 (IQR, 12–22; Range, 0–44). The prevalence of major depression (CES-D score ⩾20) was 34.6% (157/454), while 53.5% (243/454) of the participants had major depression and distress (CES-D score ⩾16) and 46.5% (211/454) had no depressive symptoms (CES-D score ⩽15). Of the 454 participants, 85.0% (386) had anal sex during the past 6 months and the rate of consistent condom use during anal intercourse was 45.1% (174/386), and 76.7% (348) had oral sex during the past 6 months and the percentage of consistent condom use during oral sex was 19.8% (69/348). Further, 32.4% reported having anal sex with more than 2 same-sex partners during the past 6 months and 31.9% engaged in oral sex during the past 6 months.

For peer review only - http://bmjopen.bmj.com/site/about/guidelines.xhtml

Suicidal ideation was reported by 16.1% (73/454) of all respondents. History of smoking was reported in 62.6% (284/454) of the participants, and 41.4% (188/454) had a history of drinking alcohol of which 46.3% (87/188) indicated they were once drunk. Only 3.7% (17/454) had excessively used drugs. Regarding sexual victimization, 7.9% (36/454) had suffered adult sexual violence from same-sex partners and 5.1% (23/454) reported sexual abuse in childhood. Only 15.9% (72/454) perceived that they have possibility of HIV infection. The percentage that reported knowing about ART for HIV/AIDS was 25.8%, 48.5% had been tested for HIV in the past, and 22.7% had regular testing for HIV (Tables 2–4).

Factors associated with depressive symptoms

Results of univariate logistic regression analyses are shown in Tables 1–4. Young age, never-been-married, no consistent condom use during anal sex in the past 6 months, number of oral sex male partners in the past 6 months, being drunk once in the past year, suicidal ideation, suffering violence from same-sex partners, experiencing sexual abuse in childhood, self-perceived possibility of HIV infection, and not knowing about ART for HIV/AIDS were associated with depressive symptoms.

Results of the multivariate analyses are shown in Table 5. Depression was associated with younger age (OR = 1.85 and 2.04 for those 16–25 years and 26–35 years compared to older than 35 years, respectively); no consistent condom use during anal sex in the past 6 months (OR = 2.03; 95%CI, 1.26-3.26; *P* = .003); suicidal ideation (OR = 4.54; 95%CI, 2.54-8.12; *P* < .001); suffering violence from same-sex partners (OR = 2.48; 95%CI,

For peer review only - http://bmjopen.bmj.com/site/about/guidelines.xhtml

1.13-5.45; *P* = .024); self-perceived possibility of HIV infection (OR = 2.36; 95%CI, 1.34-4.16; *P* = .003); and not knowing about ART for HIV/AIDS (OR = 1.68; 95%CI, 1.01-2.79; *P* = .045).

Discussion

This is the first study to explore a comprehensive range of factors for their association with depressive symptoms among MSM in China. Specifically, we investigated risky sexual behaviors, HIV-related cognitions, HIV testing history, and psychosocial factors including suicidal thoughts, substance use/abuse, and sexual victimization for their relationship to depression. We found high rates of depression, unprotected sex, and suicidal ideation in our sample. Additionally, we found that suffering adult sexual violence from same-sex partners was associated with depressive symptoms.

Our study showed a high rate of depression in MSM; higher than the reported prevalence rate of depression among the general population in China of 5.3%.[21] A similar increased risk of depression has been demonstrated among MSM in the USA, Netherlands, Kenya, and South Africa.[15,25,33-35] Due to the prevalence of depression among the MSM population greatly exceeding that of the general population, we should lay emphasis on psychosocial assessment of and interventions for MSM.

There was an association between depression and UAI (i.e., inconsistent condom use during anal intercourse) in our study, in line with the study conducted by Houston et al.[32]

For peer review only - http://bmjopen.bmj.com/site/about/guidelines.xhtml

It is possible that MSM with mild to moderate depressive symptoms address their low mood by having unprotected sex, which would increase the sense of intimacy and pleasure.[36-38] Studies have shown that UAI among MSM was the predominant factor in HIV acquisition and transmission.[11,12] Therefore, mental health interventions that focus on reducing high risk sexual behaviors and improving mental health among MSM may be effective strategies for preventing HIV infection and transmission. Additionally, future HIV prevention programs should consider incorporating mental health screening and intervention strategies to improve their effectiveness in promoting sexual health.

In our study, we found a high rate of suicidal ideation and a strong association between suicidal ideation and depressive symptoms. Li et al., in his recent study conducted in Shanghai, China, argued that not feeling a sense of belonging and having little social support may explain the high rates of suicidal ideation in MSM,[39] and the lack of a sense of belonging and social support may cause mental health issues such as depressive symptoms. A longitudinal study revealed that gay, lesbian, and bisexual youth with a history of suicide attempt were found to have higher levels of depressive and anxious symptoms.[40] Conversely, MSM with higher levels of self-esteem and greater satisfaction with social support were less likely to have major depression and suicidal ideation.[41] Given the positive association between major depression and suicidal ideation, a sense of belonging and social support may serve as protector factors as well as mediate the relationship between depression and suicidality. Given the high reported rate of suicidal ideation and depression, mental health centers should provide additional training

For peer review only - http://bmjopen.bmj.com/site/about/guidelines.xhtml

to clinicians and counselors on how to care for, screen, and manage suicidality in the MSM population. Additionally, knowledge of the psychological characteristics of MSM and development of consulting skills in clinic staff and Centers for Disease Control and Prevention (CDCs) related to working with MSM is needed to address their mental health needs, prevent suicidal behavior, and prevent HIV/AIDS infection and transmission. More generally, we should call on society to eliminate discrimination of the MSM population and provide more support and care to prevent suicidal ideation and attempts.

The study revealed that sexual victimization was associated with depressive symptoms among MSM, consistent with a study by Ratner et al.[28] Sexual violence from male partners and sexual abuse in childhood can have a negative effect on the mental health of MSM, which can increase the risk of HIV infection or other sexually transmitted infections. Consequently, health counselors and professionals should respond sensitively and give more care to victims of sexual victimization. For example, clinicians should identify the prevalence of sexual victimization among MSM, ask sensitive questions in the assessment of mental health, and deconstruct prejudicial beliefs about same-sex sexual violence.

Drinking alcohol was reported by forty percent and smoking by two thirds of our sample. Both drinking alcohol and smoking history were not associated with depressive symptoms. However, research indicates a strong association between mental health issues and substance use disorders among the general population in studies of other countries.[42,43] Further research is needed to verify the association between drinking alcohol, smoking, and depressive symptoms among Chinese MSM. Given there were few

For peer review only - http://bmjopen.bmj.com/site/about/guidelines.xhtml

drug abusers in our sample and results of different studies are divergent, the finding that there was no association between drug abuse and present depressive symptoms in our study should be cautiously interpreted.

ART has turned AIDS from a usually visible death outcome into a treatable chronic condition, which contributes to reduced stigma.[40] ART has become the most effective method to delay and control the death of AIDS or AIDS-related diseases. Not knowing about ART for HIV/AIDS was associated with depressive symptomology in our study. It is possible that knowing HIV-infected individuals can receive free ART provided by the country and life can be prolonged to a normal length reduces the mental burden of HIV infection and negative mood, including self-stigma and self-accusation among MSM. A study in Botswana revealed that perceived access to ART was strongly associated with decreased odds of HIV stigma.[44] Thus, scaling up of the treatment of HIV-infected persons would benefit the mental health of the MSM population. An association between self-perceived possibility of HIV infection and current depression symptoms was found in our study, suggesting that HIV-risk perception also had some influence on the mental condition of MSM. Health promotion geared towards MSM should address risk of HIV infection and voluntary counseling and testing (VCT) in the CDCs or clinics to improve their mental health.

The occurrence of depression symptoms was also associated with sociodemographic characteristics. Our study implied a higher prevalence of depressive symptoms among younger MSM. In his study, Salomonalso indicated that younger MSM were more likely

For peer review only - http://bmjopen.bmj.com/site/about/guidelines.xhtml

to report depressive symptoms and engage in unprotected anal sex.[24] This may be because of the maturity of older MSM in which mental regulation is stronger.[45] The finding suggests more appropriate and accessible mental health services for young MSM are needed. There was a weak association between depression and being married in the bivariate analysis, which is congruent with a study conducted by Secor et al.[25] Being married was protective against depressive symptoms possibly because marriage to a woman can help MSM hide their same-sex sexual proclivities and be more easily accepted by society. However, being married to a woman was not associated with depressive symptomology in the multivariate analyses. Further research into psychosocial and sociodemographic differences among MSM with different marital statuses is necessary to fully clarify the finding.

There are some limitations to our study. First, these data were collected via self-report, and MSM might have concerns about their privacy and underreport some sensitive information in the rather homophobic and hostile environment of China. As such, this method may have resulted in an underestimation of the true prevalence of sensitive behaviors, such as unprotected sex, drug abuse, and sexual violence. Second, the causality of depressive symptoms and associated factors is not inferred due to the cross-sectional design, and the relevant information is subjected to recall bias, which may have an influence on the magnitude of associations with depression. Third, some important psychosocial factors, such as self-esteem, social support, and negative social relationships that might mediate or moderate the associations between depressive

For peer review only - http://bmjopen.bmj.com/site/about/guidelines.xhtml

symptoms and related factors were not included in the research design. Additionally, stigma related to HIV and sexual stigma are important factors associated with depression[25] and were not examined. Fourth, different measures of depressive symptoms, such as the Self-Rating Depression Scale (SDS) and Beck Depression Inventory (BDI) may have provided results that differ from our study. Thus, longitudinal research with a larger sample size, employing more in-depth and complex measures of psychosocial and behavioral variables, and including such psychosocial factors as social support and social relationships, are needed to confirm our findings.

In response to our findings, mental health and substance abuse screening and counseling should be integrated into HIV prevention and treatment programs for the MSM population in China. Mental health issues are generally not given enough emphasis in the training of HIV/AIDS counselors in China, and we have had to provide supplementary training to staff from clinics and CDCs. More comprehensive intervention strategies that combine psychological, behavioral, and social aspects are needed to address the existing mental health issues, with special attention paid to suicidality and high-risk behaviors among MSM.

Author affiliations

[1]Center for Disease Control and Prevention of Zhejiang Province, Hangzhou, Zhejiang, China

[2]Department of Epidemiology & Biostatistics, School of Medicine, Ningbo University, Ningbo, Zhejiang, China

[3]Wenzhou Center for Disease Control and Prevention, Wenzhou, Zhejiang, China

For peer review only - http://bmjopen.bmj.com/site/about/guidelines.xhtml

[4] Lucheng Center for Disease Control and Prevention of, Wenzhou, Zhejiang, China.

Contributors SX, XP and QM were involved in the study design. HW, TJ, LH, SZ, DW, ZY, HZ and DZ performed the experiments. RL, XP and SX analysed the data. RL, XP and SX wrote the paper. XP, SX and QM critically revised the manuscript. All authors read and approved the final manuscript.

Acknowledgements We would like to thank Editage [www.editage.cn] for English language editing.

Funding Key Project on Social Development among S&T Major Project of Zhejiang Province, China (2013C03047-1).

Competing interests None declared.

Patient consent Obtained.

Ethics approval The study was approved by the ethical review board of Zhejiang Provincial Center for Disease Control and Prevention.

Data sharing statement No additional data are available.

References

1. Peirce RS, Frone MR, Russell M, *et al*. A longitudinal model of social contact, social support, depression, and alcohol use. ***Health Psychol*** 2000;19:28-38.
2. Anderson AM, Ross MW, Nyoni JE, *et al*. High prevalence of stigma-related abuse among a sample of men who have sex with men in Tanzania: implications for HIV prevention. ***AIDS Care*** 2015;27:63-70.
3. Risher K, Adams D, Sithole B, *et al*. Sexual stigma and discrimination as barriers to seeking appropriate healthcare among men who have sex with men in Swaziland. ***J Int AIDS Soc*** 2013;16:18715.
4. Cochran SD, Bandiera FC, Mays VM. Sexual orientation-related differences in tobacco use and secondhand smoke exposure among US adults aged 20 to 59 years: 2003-2010 National Health and Nutrition Examination Surveys. ***Am J Public Health*** 2013;103:1837-44.
5. Cochran SD, Mays VM. Lifetime prevalence of suicide symptoms and affective disorders among men reporting same-sex sexual partners: results from NHANES III. ***Am J Public Health*** 2000;90:573-8.
6. Farmer GW, Bucholz KK, Flick LH, *et al*. CVD risk among men participating in the National Health and Nutrition Examination Survey (NHANES) from 2001 to 2010: differences by sexual minority status. ***J Epidemiol Community Health*** 2013;67:772-8.
7. Guerim LD, de Carvalho HW, Lara DR. The relationship between temperament and sexual orientation. ***J Affect Disord*** 2015;175:379-84.
8. Mimiaga MJ, O'Cleirigh C, Biello KB, *et al*. The effect of psychosocial syndemic production on 4-year HIV incidence and risk behavior in a large cohort of sexually active men who have sex with

For peer review only - http://bmjopen.bmj.com/site/about/guidelines.xhtml

men. ***J Acquir Immune Defic Syndr*** 2015;68:329-36.
9. Tucker A, Liht J, de Swardt G, *et al*. An exploration into the role of depression and self-efficacy on township men who have sex with men's ability to engage in safer sexual practices. ***AIDS Care*** 2013;25:1227-35.
10. Grov C, Rendina HJ, Ventuneac A, *et al*. HIV risk in group sexual encounters: an event-level analysis from a national online survey of MSM in the U.S. ***J Sex Med*** 2013;10:2285-94.
11. Baggaley RF, White RG, Boily MC. HIV transmission risk through anal intercourse: systematic review, meta-analysis and implications for HIV prevention. ***Int J Epidemiol*** 2010;39:1048-63.
12. Jin F, Jansson J, Law M, *et al*. Per-contact probability of HIV transmission in homosexual men in Sydney in the era of HAART. ***AIDS*** 2010;24:907-13.
13. Ministry of Health, People's Republic of China. 2012 China AIDS Response Progress Report. Beijing: Ministry of Health; 2012. http://unaids.org.cn/pics/20120614140133.pdf
14. Ma Q, Zeng S, Xia S, *et al*. Risky sexual networks and concentrated HIV epidemics among men who have sex with men in Wenzhou, China: a respondent-driven sampling study. ***BMC Public Health*** 2015;15:1246.
15. Mills TC, Paul J, Stall R, *et al*. Distress and depression in men who have sex with men: the Urban Men's Health Study. ***Am J Psychiatry*** 2004;161:278-85.
16. Liu J, Gao YH, Liang ZM, *et al*. Depressive symptoms and associated sexual behaviors among men who have sex with men in Foshan, Guangdong province [in Chinese]. ***Zhonghua Liu Xing Bing Xue Za Zhi*** 2012;33:483-7.
17. Wang Y, Zhang HB, Xu J, Zhang GG, Yang HW, Fan J. Survey of depression status among men having sex with men[in Chinese]. ***J Prev Med Inform*** 2010;26:597-600.
18. Feng Y, Wu Z, Detels R, *et al*. HIV/STD prevalence among men who have sex with men in Chengdu, China and associated risk factors for HIV infection. ***J Acquir Immune Defic Syndr*** 2010;53 Suppl 1:S74-80.
19. Zheng YJ, Xu J, Zhang HB. Anxiety, depression and high-risk behaviors among men who have sex with men [in Chinese]. ***Chinese Mental Health Journal*** 2005;19:699-701.
20. Wu J, Lu CY, Hao YT, *et al*. Psychosocial status of MSM population in Guangzhou city [in Chinese]. ***Chin J Public Health*** 2011;27:476-8.
21. Fang F, Zhang SP, Wang ZW, *et al*. Epidemiological survey of depression among residents in a community of Shanghai [in Chinese]. ***Chin J Behav Med & Brain Sci*** 2009;18:740-2.
22. Graham LF, Braithwaite K, Spikes P, et al. Exploring the mental health of black men who have sex with men. ***Community Ment Health J*** 2009;45:272-84.
23. De Santis JP, Colin JM, Provencio Vasquez E, *et al*. The relationship of depressive symptoms, self-esteem, and sexual behaviors in a predominantly Hispanic sample of men who have sex with men. ***Am J Mens Health*** 2008;2:314-21.
24. Salomon EA, Mimiaga MJ, Husnik MJ, *et al*. Depressive symptoms, utilization of mental health care, substance use and sexual risk among young men who have sex with men in EXPLORE: implications for age-specific interventions. ***AIDS Behav*** 2009;13:811-21.
25. Secor AM, Wahome E, Micheni M, *et al*. Depression, substance abuse and stigma among men who have sex with men in coastal Kenya. ***AIDS*** 2015;29 Suppl 3:S251-9.

For peer review only - http://bmjopen.bmj.com/site/about/guidelines.xhtml

26. Hirshfield S, Wolitski RJ, Chiasson MA, et al. Screening for depressive symptoms in an online sample of men who have sex with men. ***AIDS Care*** 2008;20:904-10.

27. Paul JP, Catania J, Pollack L, *et al*. Understanding childhood sexual abuse as a predictor of sexual risk-taking among men who have sex with men: The Urban Men's Health Study. ***Child Abuse Negl*** 2001;25:557-84.

28. Ratner PA, Johnson JL, Shoveller JA, *et al*. Non-consensual sex experienced by men who have sex with men: prevalence and association with mental health. ***Patient Educ Couns*** 2003;49:67-74.

29. Reisner SL, Mimiaga MJ, Bland S, *et al*. Problematic alcohol use and HIV risk among Black men who have sex with men in Massachusetts. ***AIDS Care*** 2010;22:577-87.

30. Hernandez MT, Sanchez MA, Ayala L, *et al*. Methamphetamine and cocaine use among Mexican migrants in California: the California-Mexico Epidemiological Surveillance Pilot. ***AIDS Educ Prev*** 2009;21:34-44.

31. Blashill AJ, O'Cleirigh C, Mayer KH, *et al*. Body mass index, depression and sexual transmission risk behaviors among HIV-positive MSM. ***AIDS Behav*** 2012;16:2251-6.

32. Houston E, Sandfort T, Dolezal C, *et al*. Depressive symptoms among MSM who engage in bareback sex: does mood matter? ***AIDS Behav*** 2012;16:2209-2215.

33. Sandfort TG, Bakker F, Schellevis FG, *et al*. Sexual orientation and mental and physical health status: findings from a Dutch population survey. ***Am J Public Health*** 2006;96:1119-25.

34. Stoloff K, Joska JA, Feast D, *et al*. A description of common mental disorders in men who have sex with men (MSM) referred for assessment and intervention at an MSM clinic in Cape Town, South Africa. ***AIDS Behav*** 2013;17 Suppl 1:S77-81.

35. Tucker A, Liht J, de Swardt G, *et al*. Homophobic stigma, depression, self-efficacy and unprotected anal intercourse for peri-urban township men who have sex with men in Cape Town, South Africa: a cross-sectional association model. ***AIDS Care*** 2014;26:882-9.

36. Shiu CS, Chen YC, Tseng PC, *et al*. Curvilinear relationship between depression and unprotected sexual behaviors among men who have sex with men. ***J Sex Med*** 2014;11:2466-73.

37. Golub SA, Starks TJ, Payton G, et al. The critical role of intimacy in the sexual risk behaviors of gay and bisexual men. ***AIDS Behav*** 2012;16:626-32.

38. Craft SM, Smith SA, Serovich JM, *et al*. Need fulfillment in the sexual relationships of HIV-infected men who have sex with men. ***AIDS Educ Prev*** 2005;17:217-26.

39. Li R, Cai Y, Wang Y, *et al*. Psychosocial Syndemic Associated With Increased Suicidal Ideation Among Men Who Have Sex With Men in Shanghai, China. ***Health Psychol*** 2016;35:148-56.

40. Roura M, Urassa M, Busza J, *et al*. Scaling up stigma? The effects of antiretroviral roll-out on stigma and HIV testing. Early evidence from rural Tanzania. ***Sex Transm Infect*** 2009;85:308-12.

41. Sivasubramanian M, Mimiaga MJ, Mayer KH, *et al*. Suicidality, clinical depression, and anxiety disorders are highly prevalent in men who have sex with men in Mumbai, India: findings from a community-recruited sample. ***Psychol Health Med*** 2011;16:450-62.

42. King M, Semlyen J, Tai SS, *et al*. A systematic review of mental disorder, suicide, and deliberate self harm in lesbian, gay and bisexual people. ***BMC Psychiatry*** 2008;8:70.

43. Schneider B, Kolves K, Blettner M, *et al*. Substance use disorders as risk factors for suicide in an Eastern and a Central European city (Tallinn and Frankfurt/Main). ***Psychiatry Res***

For peer review only

2009;165:263-72.
44. Wolfe WR, Weiser SD, Leiter K, *et al*. The impact of universal access to antiretroviral therapy on HIV stigma in Botswana. ***Am J Public Health*** 2008;98:1865-71.
45. Kipke MD, Kubicek K, Weiss G, *et al*. The health and health behaviors of young men who have sex with men. ***J Adolesc Health*** 2007;40:342-50.

Table 1. Sociodemographic characteristics and their associations with depression among MSM in Wenzhou, China (N = 454)

**Variables**	**Total n(%)**	**Major Depression n(%)**	**OR (95%CI)**	***P* value**
**Age(years)** (median = 33; IQR, 26–41)				
≤25	101(22.2)	39(38.6)	1.70(1.01-2.84)	0.044
26–35	168(37.0)	68(40.5)	1.84(1.17-2.87)	0.008
36 and older	185(40.7)	50(27.0)	1.00	
**Marital status**				
Never married	253(55.7)	97(38.3)	1.00	
Married/Divorced/Widowed	201(44.3)	60(29.9)	0.68(0.46-0.99)	0.049
**Education level**				
Junior school or lower	272(59.9)	87(32.0)	1.00	
Senior school	121(26.7)	50(41.3)	1.50(0.96-2.33)	0.074
College or higher	61(13.4)	20(32.8)	1.04(0.57-1.88)	0.904
**Monthly income (RMB)**				
<3000	194(42.7)	62(32.0)	1.00	
3000 and above	260(57.3)	95(36.5)	1.23(0.83-1.82)	0.310
**Duration of residence in Wenzhou (years)**				
≤3	220(48.6)	79(35.9)	1.00	
>3	233(51.4)	77(33.0)	0.88(0.60-1.30)	0.522
**Insurance**				

For peer review only

For peer review only - http://bmjopen.bmj.com/site/about/guidelines.xhtml

Insured	140(30.8)	54(38.6)	1.00	
Uninsured	314(69.2)	103(32.8)	0.78(0.51-1.18)	0.233
**Sexual orientation**				
Homosexual	184(40.5)	71(38.6)	1.34(0.91-1.99)	0.139
Heterosexual or bisexual or indeterminate	270(59.5)	86(31.9)	1.00	
**Places to seek for sexual partners**				
Venues	261(59.9)	85(32.6)	1.00	
Internet	175(40.1)	66(37.7)	1.25(0.84-1.87)	0.269
**Number of MSM which a participant knew in his circle of MSM friends locally** (median = 7; IQR, 3–15)				
>7	220(48.5)	74(33.6)	0.92(0.63-1.36)	0.681
≤7	234(51.5)	83(35.5)	1.00	

MSM = men who have sex with men, OR = odds ratio, CI = confidence interval, IQR = interquartile range.

Table 2. Sexual behavior and their associations with depression among MSM in Wenzhou, China (N = 454)

**Variables**	**Total n(%)**	**Major Depression n(%)**	**OR (95%CI)**	***P* value**
**Age of sexual debut with a man** (median = 24; IQR, 20–30)				
≤22	197(43.4)	70(35.5)	1.08(0.73-1.59)	0.709
>22	257(56.6)	87(33.9)	1.00	
**Anal intercourse with men in the past 6 months**				
Yes	386(85.0)	139(36.0)	1.56(0.88-2.79)	0.129
No	68(15.0)	18(26.5)	1.00	
**Number of anal intercourse male partners in the past 6 months**				

For peer review only - http://bmjopen.bmj.com/site/about/guidelines.xhtml

For peer review only

(median = 2; IQR, 1–3)				
≤2	307(67.6)	98(31.9)	1.00	
>2	147(32.4)	59(40.1)	1.43(0.95-2.15)	0.086
**Consistent condom use during anal sex in the past 6 months**				
Yes	174(45.1)	46(26.4)	1.00	
No	212(54.9)	93(43.9)	2.18(1.41-3.35)	<0.001
**Oral sex with men in the past 6 months**				
Yes	348(76.7)	124(35.6)	1.23(0.77-1.95)	0.394
No	106(23.3)	33(31.1)	1.00	
**Number of oral sex male partners in the past 6 months**				
≤2	309(68.1)	97(31.4)	1.00	
>2	145(31.9)	60(41.4)	1.54(1.03-2.32)	0.038
**Consistent condom use during oral sex in the past 6 months**				
Yes	69(19.8)	24(34.8)	1.00	
No	279(80.2)	100(35.8)	1.05(0.60-1.82)	0.869
**Sex with women in the past**				
Yes	348(76.7)	115(33.0)	0.75(0.48-1.18)	0.213
No	106(23.3)	42(39.6)	1.00	
**Age of sexual debut with a woman**				
≤22	209(60.1)	71(34.0)	1.11(0.70-1.76)	0.653
>22	139(39.9)	44(31.7)	1.00	
**Sex with women in the past 6 months**				
Yes	167(36.8)	59(35.3)	1.05(0.71-1.57)	0.798
No	287(63.2)	98(34.1)	1.00	
**Number of female sex partners in the past 6 months**				
<2	407(90.0)	137(33.7)	1.00	
≥2	45(10.0)	19(42.2)	1.44(0.77-2.69)	0.254

For peer review only - http://bmjopen.bmj.com/site/about/guidelines.xhtml

**Consistent condom use during sex with women in the past 6 months**				
Yes	53(31.7)	21(39.6)	1.00	
No	114(68.3)	38(33.3)	0.76(0.39-1.50)	0.429

MSM = men who have sex with men, OR = odds ratio, CI = confidence interval, IQR = interquartile range.

Table 3. Psychosocial factors and their associations with depression among MSM in Wenzhou, China

(N = 454)

**Variables**	**Total n(%)**	**Major Depression n(%)**	**Odds Ratio (95%CI)**	***P* value**
**Smoking**				
Yes	284(62.6)	98(34.5)	0.99(0.67-1.48)	0.966
No	170(37.4)	59(34.7)	1.00	
**Drinking alcohol**				
Yes	188(41.4)	64(34.0)	0.96(0.65-1.42)	0.839
No	266(58.6)	93(35.0)	1.00	
**Being drunk once in the past year**				
Yes	87(46.3)	39(44.8)	2.47(1.33-4.59)	0.004
No	101(53.7)	25(24.8)	1.00	
**Wanting or needing to reduce drinking in the past year**				
Yes	48(55.2)	26(54.2)	2.36(0.99-5.67)	0.054
No	39(44.8)	13(33.3)	1.00	
**Drug abuse**				
Yes	17(3.7)	8(47.1)	1.72(0.65-4.55)	0.275

For peer review only - http://bmjopen.bmj.com/site/about/guidelines.xhtml

No	437(96.3)	149(34.1)	1.00	
**Suicidal ideation**				
Yes	73(16.1)	50(68.5)	5.57(3.24-9.57)	<0.001
No	381(83.9)	107(28.1)	1.00	
**Adult sexual violence from same-sex partners**				
Yes	36(7.9)	23(63.9)	3.75(1.84-7.63)	<0.001
No	418(92.1)	134(32.1)	1.00	
**Childhood sexual abuse**				
Yes	23(5.1)	14(60.9)	3.13(1.32-7.41)	0.009
No	431(94.9)	143(33.2)	1.00	

MSM = men who have sex with men, OR = odds ratio, CI = confidence interval.

Table 4. HIV related cognition and testing history and their associations with depression among MSM in Wenzhou, China (N = 454)

**Variables**	**Total n(%)**	**Major Depression n(%)**	**Odds Ratio (95%CI)**	***P* value**
**Influence of social perception on sexual behaviors of MSM population**				
Yes	239(52.6)	92(38.5)	1.44(0.98-2.14)	0.065
No	215(47.4)	65(30.2)	1.00	
**Self-perceived possibility of HIV infection**				
Possible	72(15.9)	41(56.9)	3.03(1.81-5.08)	<0.001
Impossible/unlikely	382(84.1)	116(30.4)	1.00	
**Estimate of HIV prevalence among MSM**				
≤10%	301(66.3)	95(31.6)	1.00	
>10%	153(33.7)	62(40.5)	1.48(0.99-2.21)	0.058
**Exposure to AIDS/STDs propaganda intervention during the previous year**				

For peer review only - http://bmjopen.bmj.com/site/about/guidelines.xhtml

No	200(44.1)	68(34.0)	1.00	
Yes	254(55.9)	89(35.0)	1.05(0.71-1.55)	0.817
**Knowing about ART for HIV/AIDS**				
Yes	117(25.8)	31(26.5)	1.00	0.034
No	337(74.2)	126(37.4)	1.67(1.04-2.63)	
**Self-perceived necessity to accept early ART for HIV-positive people**				
Yes	431(94.9)	149(34.6)	0.99(0.41-2.39)	0.983
No	23(5.1)	8(34.8)	1.00	
**Prior HIV testing**				
No	220(48.5)	76(34.5)	1.00	
Yes	234(51.5)	81(34.6)	1.00(0.68-1.48)	0.988
**HIV testing in the past year**				
No	256(56.4)	89(34.8)	1.00	
Yes	198(43.6)	68(34.3)	0.98(0.66-1.45)	0.925
**Regular testing for HIV**				
Yes	103(22.7)	39(37.9)	1.20(0.76-1.90)	0.426
No	351(77.3)	118(33.6)	1.00	
**Intention for regular HIV testing**				
Yes	384(84.6)	132(34.4)	0.94(0.55-1.61)	0.828
No	70(15.4)	25(35.7)	1.00	

MSM = men who have sex with men, OR = odds ratio, CI = confidence interval, HIV = human immunodeficiency virus, AIDS = acquired immune deficiency syndrome, STDs = sexually transmitted diseases, ART = antiretroviral therapy.

Table 5. Multivariate analyses of factors associated with depression among MSM in Wenzhou, China

(N = 454)

For peer review only - http://bmjopen.bmj.com/site/about/guidelines.xhtml

Variable	AOR(95%CI)	*P* value
**Age(years)**		
≤25	1.85(1.05-3.26)	0.034
26–35	2.04(1.24-3.35)	0.005
36 and older	1.00	
**Consistent condom use during anal sex in the past 6 months**		
Yes	1.00	
No	2.03(1.26-3.26)	0.003
**Suicidal ideation**		
No	1.00	
Yes	4.54(2.54-8.12)	<0.001
**Adult sexual violence from male sexual partners**		
No	1.00	
Yes	2.48(1.13-5.45)	0.024
**Self-perceived possibility of HIV infection**		
Possible	2.36(1.34-4.16)	0.003
Impossible/unlikely	1.00	
**Knowing about ART for HIV/AIDS**		
Yes	1.00	
No	1.68(1.01-2.79)	0.045

MSM = men who have sex with men, AOR = adjusted odds ratio, CI = confidence interval, HIV = human immunodeficiency virus, AIDS = acquired immune deficiency syndrome, ART = antiretroviral therapy.

For peer review only

STROBE 2007 (v4) Statement—Checklist of items that should be included in reports of *cross-sectional studies*

Section/Topic	Item #	Recommendation	Reported on page #
**Title and abstract**	1	(*a*) Indicate the study's design with a commonly used term in the title or the abstract	1-2
		(*b*) Provide in the abstract an informative and balanced summary of what was done and what was found	2-3
**Introduction**			
Background/rationale	2	Explain the scientific background and rationale for the investigation being reported	4-5
Objectives	3	State specific objectives, including any prespecified hypotheses	5
**Methods**			
Study design	4	Present key elements of study design early in the paper	5-6
Setting	5	Describe the setting, locations, and relevant dates, including periods of recruitment, exposure, follow-up, and data collection	5-7
Participants	6	(*a*) Give the eligibility criteria, and the sources and methods of selection of participants	6
Variables	7	Clearly define all outcomes, exposures, predictors, potential confounders, and effect modifiers. Give diagnostic criteria, if applicable	7-9
Data sources/ measurement	8*	For each variable of interest, give sources of data and details of methods of assessment (measurement). Describe comparability of assessment methods if there is more than one group	7-9
Bias	9	Describe any efforts to address potential sources of bias	
Study size	10	Explain how the study size was arrived at	6
Quantitative variables	11	Explain how quantitative variables were handled in the analyses. If applicable, describe which groupings were chosen and why	9
Statistical methods	12	(*a*) Describe all statistical methods, including those used to control for confounding	9
		(*b*) Describe any methods used to examine subgroups and interactions	
		(*c*) Explain how missing data were addressed	
		(*d*) If applicable, describe analytical methods taking account of sampling strategy	9
		(*e*) Describe any sensitivity analyses	
**Results**			

For peer review only

Participants	13*	(a) Report numbers of individuals at each stage of study—eg numbers potentially eligible, examined for eligibility, confirmed eligible, included in the study, completing follow-up, and analysed	10
		(b) Give reasons for non-participation at each stage	
		(c) Consider use of a flow diagram	
Descriptive data	14*	(a) Give characteristics of study participants (eg demographic, clinical, social) and information on exposures and potential confounders	10-11
		(b) Indicate number of participants with missing data for each variable of interest	
Outcome data	15*	Report numbers of outcome events or summary measures	10-11
Main results	16	(*a*) Give unadjusted estimates and, if applicable, confounder-adjusted estimates and their precision (eg, 95% confidence interval). Make clear which confounders were adjusted for and why they were included	11-12
		(*b*) Report category boundaries when continuous variables were categorized	
		(*c*) If relevant, consider translating estimates of relative risk into absolute risk for a meaningful time period	
Other analyses	17	Report other analyses done—eg analyses of subgroups and interactions, and sensitivity analyses	
**Discussion**			
Key results	18	Summarise key results with reference to study objectives	12
Limitations	19	Discuss limitations of the study, taking into account sources of potential bias or imprecision. Discuss both direction and magnitude of any potential bias	16-17
Interpretation	20	Give a cautious overall interpretation of results considering objectives, limitations, multiplicity of analyses, results from similar studies, and other relevant evidence	12-16
Generalisability	21	Discuss the generalisability (external validity) of the study results	
**Other information**			
Funding	22	Give the source of funding and the role of the funders for the present study and, if applicable, for the original study on which the present article is based	18

*Give information separately for cases and controls in case-control studies and, if applicable, for exposed and unexposed groups in cohort and cross-sectional studies.

Note: An Explanation and Elaboration article discusses each checklist item and gives methodological background and published examples of transparent reporting. The STROBE checklist is best used in conjunction with this article (freely available on the Web sites of PLoS Medicine at http://www.plosmedicine.org/, Annals of Internal Medicine at http://www.annals.org/, and Epidemiology at http://www.epidem.com/). Information on the STROBE Initiative is available at www.strobe-statement.org.

BMJ Open

Sexual risk behavior, sexual victimization, substance use, and other factors related to depression in men who have sex with men in Wenzhou, China: a cross-sectional study

Journal:	*BMJ Open*
Manuscript ID	bmjopen-2016-013512.R1
Article Type:	Research
Date Submitted by the Author:	24-Oct-2016
Complete List of Authors:	Pan, Xiaohong; Center for Disease Control and Prevention of Zhejiang Province<br>Li, Runhua; Ningbo University, Medical school<br>Ma, Qiaoqin; Center for Disease Control and Prevention of Zhejiang Province, ;<br>Wang, Hui; Center for Disease Control and Prevention of Zhejiang Province,<br>Jiang, Tingting; Center for Disease Control and Prevention of Zhejiang Province,<br>He, Lin; Center for Disease Control and Prevention of Zhejiang Province,<br>Zeng, Shidian; Wenzhou Center for Disease Control and Prevention,<br>Wang, Dayong; Wenzhou Center for Disease Control and Prevention,<br>Ye, Zhenmiao; Wenzhou Center for Disease Control and Prevention,<br>Zhu, Haishen; Center for Disease Control and Prevention of Lucheng District, Wenzhou<br>Zhao, Dongshe; Lucheng Center for Disease Control and Prevention<br>Xia, Shichang; Center for Disease Control and Prevention of Zhejiang Province,
**Primary Subject Heading**:	Public health
Secondary Subject Heading:	HIV/AIDS, Mental health
Keywords:	PUBLIC HEALTH, HIV & AIDS < INFECTIOUS DISEASES, MENTAL HEALTH

SCHOLARONE™
Manuscripts

Sexual risk behavior, sexual victimization, substance use, and other factors related to depression in men who have sex with men in Wenzhou, China: a cross-sectional study

#Xiaohong Pan[1], #Runhua Li[1,2], Qiaoqin Ma[1], Hui Wang[1], Tingting Jiang[1], Lin He[1], Shidian Zeng[3], Dayong Wang[3], Zhenmiao Ye[3], Haishen Zhu[4], Dongshe Zhao[4], *Shichang Xia[1]

[1]Center for Disease Control and Prevention of Zhejiang Province, Hangzhou, Zhejiang, China

[2]Department of Epidemiology & Biostatistics, School of Medicine, Ningbo University, Ningbo, Zhejiang, China

[3]Wenzhou Center for Disease Control and Prevention, Wenzhou, Zhejiang, China

[4] Lucheng Center for Disease Control and Prevention, Wenzhou, Zhejiang, China.

*Corresponding author: Shichang Xia

Email: shchxia@cdc.zj.cn

#Xiaohong Pan, and Runhua Li contributed equally to this work.

For peer review only - http://bmjopen.bmj.com/site/about/guidelines.xhtml

Abstract

Objectives: Men who have sex with men (MSM) are a marginalized population at high risk for a variety of mental health problems that may mutually facilitate HIV transmission. We assessed factors associated with depressive symptoms among MSM, which can provide some guidance for future mental health interventions with the MSM population for prevention of HIV infection and transmission. **Methods:** A cross-sectional study was conducted in Wenzhou City, China using respondent-driven sampling between December 2013 and June 2014. A face-to-face questionnaire survey was employed to collect information about mental and psychosocial health conditions and sexual behavior characteristics among MSM. Bivariate and multivariate logistic regression analyses were used to identify factors associated with major depressive symptoms. **Results:** Of the 454 participants in the study, prevalence of major depressive symptoms was 34.6%. During the past 6 months, 85% had anal sex with men, and rate of consistent condom use during anal intercourse was 45.1%. Of the respondents, 16.1% reported suicidal ideation, 62.6% had a history of smoking, and 41.4% had a history of drinking alcohol, of which 46.3% reported that they were once drunk. Drug use was reported in 3.7% of the sample. Adult sexual violence from same-sex partners was 7.9%, and 5.1% reported childhood sexual abuse. A RDS-weighted multivariate analysis showed that major depressive symptoms among MSM were associated with inconsistent condom use during anal sex with men; multiple oral male sexual partners； suicidal ideation; adult sexual violence from male partners; and being once drunk in the past year. **Conclusion:** We found high levels of

For peer review only - http://bmjopen.bmj.com/site/about/guidelines.xhtml

depressive symptoms, unprotected sex, and suicidal ideation among MSM. Comprehensive intervention strategies that combine psychological, behavioral, and social aspects are needed to address the mental health issues of MSM, with special attention given to suicidality and high-risk behaviors in mental health and HIV prevention interventions.

Strengths and limitations of this study

1. The present study obtained the prevalence of major depressive symptoms, sexual risk behaviors, sexual victimization, suicidal ideation, substance use and elucidated some related syndemic factors to depressive symptoms among MSM in Wenzhou with high HIV prevalence.

2. Our study can provide some guidance for mental health and HIV prevention interventions for the MSM population.

3. It is unavoidable that MSM might underreport some sensitive issues such as unprotected sex, drug abuse, and sexual violence covered in the questionnaire in the rather homophobic and hostile environment of China.

4. The causality of depressive symptoms and associated risk factors is not inferred due to the cross-sectional design, and the relevant information is subjected to recall bias, which may have an influence on the magnitude of associations with depression.

For peer review only

For peer review only - http://bmjopen.bmj.com/site/about/guidelines.xhtml

Introduction

Men who have sex with men (MSM) are a marginalized population facing many challenges including stigma, abuse, social and legal discrimination, and lack of social support,[1-3] which may result in high risk for a variety of mental and psychosocial health problems, such as depression, suicidality, and drug and alcohol abuse.[4-7] A large prospective cohort study showed that syndemics of depressive symptoms, heavy alcohol use and drug use, and childhood sexual abuse (CSA) increased HIV risk and HIV-related risk behaviors.[8] Depression and substance abuse among MSM have been linked to condomless anal intercourse (CAI),[9,10] which was the predominant factor of HIV acquisition and transmission.[11,12] These interactive factors have resulted in high HIV prevalence and incidence among MSM. It is estimated that MSM accounted for 29.4 % of the new HIV infections in China in 2011.[13] In Wenzhou City of China, the prevalence of HIV was estimated to be 22.8% (95% confidence interval [CI], 16.9 to 28.5%) in a previous publication.[14]

A survey conducted in four large American cities showed the prevalence of depressive symptoms among MSM was 29.2%, nearly three times that of the general population.[15] In China, the prevalence of depressive symptoms among MSM has varied from 34.8% to 63.9%,[16-20] which was much higher than 5.3% among the general population.[21] Previous studies showed that factors associated with depression among MSM included race,[22] lower educational level,[23] younger age,[24] and marriage to a

For peer review only

woman.[25,26] Psychological factors such as sexual victimization,[27,28] substance abuse,[29,30] and HIV or sexual stigma[25] were associated with depressive symptoms among MSM. Some studies showed that engaging in high-risk sexual behaviors was associated with depressive symptoms among the MSM population.[31,32] In China, homosexual behavior has been subject to prejudice because it continues to be seen by many people as a rejection of China's cultural tradition to marry and have children.[33,34] The marriage between two men was also not admitted in Chinese laws. Chinese MSM have suffered much social stigma and discrimination from the whole society due to their sexual orientation.[33,35,36] Prior research suggests that the pressures tied to maintaining their needs of homosexual behavior and culturally normative social duties are associated with stress and internalized feelings of blame and low self-worth among Chinese MSM.[36,37] Minority stress theory has been proposed to explain the well-documented prevalence of various mental health problems, such as depression and anxiety symptoms, and suicidal thoughts among MSM.[38]

Few studies have examined the associations between depressive symptoms among MSM and sexual risk behaviors, sexual victimization, and substance use, which is very important to guide mental health and HIV interventions among MSM in China. These previous studies were conducted to examine several independent factors associated with depression and did not comprehensively consider integrating sociodemographic characteristics, high-risk sexual behaviors, psychosocial factors and substance use into their analyses.

For peer review only

Some studies conducted in the US and in European countries examined depression and associated factors among MSM, while such research in China has been limited and lacks comprehensiveness. In the present study, our endeavor was to ascertain the prevalence of depression in Wenzhou City, China and related syndemic factors, including sexual risk behaviors, suicidality, sexual victimization, and substance use. Such knowledge would provide guidance for mental health and HIV prevention interventions for the MSM population.

Materials and methods

Study population and sampling

This cross-sectional study was conducted in Wenzhou City, China between December 2013 and June 2014. This baseline survey research was conducted for the purposes of controlling HIV/AIDS new infections among the MSM population in Zhejiang Province, China. The study procedures and sampling strategies are described in detail elsewhere.[14] Briefly, participants were recruited using a respondent−driving sampling (RDS) method. Participants who were at least 16 years old, had resided in Wenzhou at least 3 months, self-reported having had anal sex and/or oral sex in the past 12 months, and were willing to participate in the survey met inclusion criteria in this study. First, we selected 5 initial active “seed” participants meeting inclusion criteria from the local MSM population. After completing the field investigation and relevant testing, “seed” participants were provided with 3 recruitment coupons, and through the

For peer review only - http://bmjopen.bmj.com/site/about/guidelines.xhtml

incentive measures the "seed" participants then referred a maximum of 3 respondents from their social network to participate in the survey. These respondents continued to refer a maximum of 3 peers to the study and continued to recruit MSM participants until equilibrium was reached. The sample composition was considered to have reached equilibrium when their socio-demographic characteristics were stable, and would not change much for further recruitment of participants (the key socio-demographic characteristics such as age, marital status, education level, monthly income and sexual orientation changed <2% in a subsequent wave recruitment of peers compared with the previous one), irrespective of the characteristics of the initial seeds. All participants received remuneration in the amount of RMB 100 (approximately US$ 16) for participation and another RMB 100 as an incentive to refer peers to participate in the study. Finally, 5 seeds recruited 30, 61, 81, 103, and 174 MSM, respectively, with a total sample of 454 MSM by 14 waves of recruitment. All eligible participants were interviewed by trained staff from the Center for Disease Control and Prevention of Lucheng District, Wenzhou, who also collected the questionnaire about sociodemographics, sexual risk behaviors, psychosocial conditions, HIV-related cognitions, and HIV testing history of participants.

Measures

Mental and psychosocial health conditions

Depression. Depressive symptoms were assessed using the Center for Epidemiological Studies Depression Scale (CES-D). The CES-D consists of 20 items

For peer review only - http://bmjopen.bmj.com/site/about/guidelines.xhtml

designed to assess the experience of depression. Respondents indicate the extent to which each item statement describes their experience during the past week. Response options include the following: “rarely or none of the time (less than 1 day),” “some or a little of the time (1–2 days),” “occasionally or a moderate amount of the time (3–4 days),” and “most or all of the time (5–7 days).” Responses are on a 4-point Likert sale and scored 0–3. Level of depression was categorized as follows: major depressive symptoms (CES-D score of 20 or higher), moderate depressive symptoms (CES-D score of 16–19), and no depressive symptoms (CES-D score of 15 or lower). Thus, higher scores indicate higher levels of depressive symptoms. A total scale score of 20 or higher was used to define major depressive symptoms as the main analysis variable in our study.

Sexual victimization. Sexual victimization included childhood sexual abuse and adult sexual violence from same-sex partners. We defined childhood sexual abuse as the experience of being “forced or frightened by someone into doing something sexually” with a partner more than 10 years older than the respondent when the respondent was aged 15 years or younger.[39] Those respondents who reported that they were first forced or coerced to have sex against their will by men when 16 years of age or older were classified as having experienced adult sexual violence. These respondents did not report experiencing childhood sexual abuse (i.e. when under the age of 16 years).[40,41]

Suicidal ideation. Participants were asked: “during the past year, have you ever had thoughts of taking your own life?” The definition of suicidal ideation referred to previous research.[42]

For peer review only - http://bmjopen.bmj.com/site/about/guidelines.xhtml

Use of substances

Substance use included smoking cigarettes, alcohol consumption, and use of drugs. Cigarette smoking refers to smoking one or more cigarettes every day for more than a year or smoking more than 300 cigarettes in 3 months or less. Alcohol consumption refers to ethanol intake of 100 grams per week such as 250 grams of 40% volume of wine consumption or 1 kilogram of 10% volume of red wine intake. According to the definition, participants were asked whether they had a history of smoking and drinking alcohol, whether they had been drunk in the past year, and whether they wanted or needed to reduce drinking in the past year. Drug use were defined as using at least two kinds of the following drugs at least monthly during the past 12 months: sedatives, morphine, mescalinum, LSD, opiates, cocaine, marijuana, heroin, ecstasy, other street or club drugs.[43]

Sexual risk behaviors

Sexual risk behaviors included 6 items: 1) number of anal intercourse male partners in the past 6 months, 2) consistent condom use during anal sex with men, 3) number of oral sex with male partners in the past 6 months, 4) consistent condom use during oral sex with men, 5) number of female sexual partners, and 6) consistent condom use during vaginal intercourse with women.

Translation, validation and reliablility

Measures were independently translated from English to Chinese by two staff members fluent in both languages. The surveys were then back-translated into English to

For peer review only - http://bmjopen.bmj.com/site/about/guidelines.xhtml

ensure that they retained their original meaning and good validation of the translation. The Cronbach's alpha coefficient of the CES-D in a large population from 39 cities in China was 0.90.[44] The Cronbach' alpha coefficient of the CES-D was 0.88 for this sample. The scale is fit for Chinese population with good validation and reliability. [44,45]

Statistical analysis

Data were entered into EpiData version 3.1 (http://www.epidata.dk/) via double entry. After data cleaning and verification, the data were processed using PASW Statistics 18.0 (SPSS Inc., Chicago, IL, USA) for statistical analysis. For descriptive analyses, the mean, standard deviation (SD), median, and interquartile range (IQR) were computed for continuous variables and frequencies for categorical variables. Multicollinearity was diagnosed by examining the variance inflation factors (VIF). Variables significantly associated with major depressive symptoms at the level of $P < .05$ in the univariate logistic analyses were included in the multivariate logistic regression model, controlling for demographic variables (age, marital status, education level, monthly income, and sexual orientation) and calculating adjusted odds ratios (AOR) and 95% confidence intervals (CI). Then, we used the Respondent-Driven Sampling Analysis Tool (RDSAT) version 7.1 (http://www.respondentdrivensampling.org/) to produce individualized weights for adjusting data of different participants with different network sizes.[46-48] The individualized weights were imported into PASW Statistics 18.0 to conduct weighted multivariate logistic regression analyses. Statistical significance was defined as $P < .05$.

Ethics statement

For peer review only - http://bmjopen.bmj.com/site/about/guidelines.xhtml

The study was approved by the ethical review board of Zhejiang Provincial Center for Disease Control and Prevention. No harm was given to the participants by the study and we protected confidentiality of the participants. A written informed consent was signed by all participants during the survey.

Results

Sample characteristics

A total of 454 MSM participated in the study, and the characteristics of the sample are provided in Table 1. Of the 454 participants, 55.7% (253/454) were never married and 51.4% (233/453) resided in Wenzhou City for more than three years. The age of the respondents ranged from 17 to 76 years (median = 33; IQR, 26–41). Regarding education level, 86.6% (393/454) had an education of senior school or lower. Roughly sixty percent (57.3%) of the participants had an income of not less than 3000 RMB monthly, 30.8% (140/454) had health insurance, and 40.5% reported their sexual orientation as homosexual or gay(Table 1).

Depressive symptoms, sexual risk behaviors, suicidal ideation, substance use, and sexual victimization

The median CES-D scale score was 16 (IQR, 12–22; Range, 0–44). The prevalence of major depressive symptoms (CES-D score ⩾20) was 34.6% (157/454), while 53.5% (243/454) of the participants had moderate to major depressive symptoms (CES-D score ⩾16) and 46.5% (211/454) had no depressive symptoms (CES-D score ⩽15). Of the 454 participants, 85.0% (386/454) had anal sex with men during the past 6 months and

For peer review only

the rate of consistent condom use during anal intercourse was 45.1% (174/386), and 76.7% (348/454) had oral sex with men during the past 6 months and the percentage of consistent condom use during oral sex was 19.8% (69/348). Further, 32.4% reported having anal sex with more than 2 same-sex partners during the past 6 months and 31.9% engaged in oral sex during the past 6 months. Suicidal ideation was reported by 16.1% (73/454) of all respondents. History of smoking was reported in 62.6% (284/454) of the participants, and 41.4% (188/454) had a history of drinking alcohol of which 46.3% (87/188) indicated they were once drunk. Only 3.7% (17/454) had used drugs. Regarding sexual victimization, 7.9% (36/454) had suffered adult sexual violence from same-sex partners and 5.1% (23/454) reported sexual abuse in childhood. (Table 2–3).

Factors associated with depressive symptoms

Results of univariate logistic regression analyses are shown in Tables 1–4. Young age, never-been-married, no consistent condom use during anal sex in the past 6 months, number of oral sex male partners in the past 6 months, being once drunk in the past year, suicidal ideation, suffering violence from same-sex partners, and experiencing sexual abuse in childhood were associated with depressive symptoms.

Results of the RDS-weighted multivariate analyses are shown in Table 4. Major depressive symptoms were associated with inconsistent condom use during anal sex in the past 6 months (AOR = 3.62; 95%CI, 2.15-6.13; $P < .001$); more than two oral sexual male partners in the past 6 months (AOR = 1.75; 95%CI, 1.09-2.80; $P = .020$); suicidal ideation (AOR = 5.09; 95%CI, 2.59-9.97; $P < .001$); suffering adult violence from

For peer review only

same-sex partners (AOR = 2.57; 95%CI, 1.03-6.73; *P* = 0.031); being once drunk in the past year (AOR = 2.89; 95%CI, 1.36-6.14; *P* = .006).

Discussion

This is the first study to explore a comprehensive range of factors for their association with depressive symptoms among MSM in China. Specifically, we investigated risky sexual behaviors, substance use, and psychosocial factors including suicidal thoughts, and sexual victimization for their relationship to depressive symptoms. We found high rates of depressive symptoms, unprotected sex, and suicidal ideation in our sample. Additionally, we found that suffering adult sexual violence from same-sex partners was associated with depressive symptoms.

Our study showed a high rate of depressive symptoms in MSM; higher than the reported prevalence rate of depressive symptoms among the general population in China of 5.3%.[21] A similar increased risk of depressive symptoms has been demonstrated among MSM in the USA, Netherlands, Kenya, and South Africa.[15,25,49-51] Due to the prevalence of depressive symptoms among the MSM population greatly exceeding that of the general population, we should lay emphasis on psychosocial assessment of and interventions for MSM.

It is noteworthy to have an association between depressive symptoms and CAI (i.e., inconsistent condom use during anal intercourse) in our study, in line with the study conducted by Houston et al.[32] It is possible that MSM with mild to moderate depressive

For peer review only - http://bmjopen.bmj.com/site/about/guidelines.xhtml

symptoms address their low mood by having unprotected sex, which would increase the sense of intimacy and pleasure.[52-54] Studies have shown that CAI among MSM was the predominant factor in HIV acquisition and transmission.[11,12] Of particular concern were the rising incidence and high prevalence of HIV and lower rate of condom use when engaging in anal sex among MSM in China, specially in Wenzhou.[14,55] Therefore, mental health interventions that focus on reducing high risk sexual behaviors and improving mental health among MSM may be effective strategies for preventing HIV infection and transmission. Additionally, future HIV prevention programs should consider incorporating mental health screening and intervention strategies to improve their effectiveness in promoting sexual health.

In our study, we found a high rate of suicidal ideation and a strong association between suicidal ideation and depressive symptoms. Li et al., in his recent study conducted in Shanghai, China, argued that not feeling a sense of belonging and having little social support may explain the high rates of suicidal ideation in MSM,[-56] and the lack of a sense of belonging and social support may cause mental health issues such as depressive symptoms. A longitudinal study revealed that lesbian, gay, bisexual, andtransgender (LGBT) youth with a history of suicide attempt were found to have higher levels of depressive and anxious symptoms.[57] Conversely, MSM with higher levels of self-esteem and greater satisfaction with social support were less likely to have major depression and suicidal ideation.[58] Given the positive association between major depression and suicidal ideation, a sense of belonging and social support may serve as

For peer review only - http://bmjopen.bmj.com/site/about/guidelines.xhtml

protector factors as well as mediate the relationship between depression and suicidality. Given the high reported rate of suicidal ideation and depression, mental health centers should provide additional training to clinicians and counselors on how to care for, screen, and manage suicidality in the MSM population. Additionally, knowledge of the psychological characteristics of MSM and development of consulting skills in clinic staff and Centers for Disease Control and Prevention (CDCs) related to working with MSM is needed to address their mental health needs, prevent suicidal behavior, and prevent HIV/AIDS infection and transmission. More generally, we should call on society to eliminate discrimination of the MSM population and provide more support and care to prevent suicidal ideation and attempts.

The study revealed that sexual victimization was associated with depressive symptoms among MSM, consistent with a study by Ratner et al.[28] Sexual violence from male partners and sexual abuse in childhood can have a negative effect on the mental health of MSM, which can increase the risk of HIV infection or other sexually transmitted infections. Consequently, health counselors and professionals should respond sensitively and give more care to victims of sexual victimization. For example, clinicians should identify the prevalence of sexual victimization among MSM, ask sensitive questions in the assessment of mental health, and deconstruct prejudicial beliefs about same-sex sexual violence.

Drinking alcohol was reported by forty percent and smoking by two thirds of our sample. Both drinking alcohol and smoking history were not associated with depressive symptoms. However, research indicates a strong association between mental health issues

For peer review only - http://bmjopen.bmj.com/site/about/guidelines.xhtml

and substance use disorders among the general population in studies of other countries.[59-60] Our study only indicated that being once drunk were associated with depressive symptoms among MSM. Further research is needed to verify the association between drinking alcohol, smoking, and depressive symptoms among Chinese MSM. Given there were few drug users in our sample and results of different studies are divergent, the finding that there was no association between drug use and present depressive symptoms in our study should be cautiously interpreted.

Previous studies have shown that high levels of sexual stigma can lead to internalized homophobia, and poor social support, all of which are risk factors for mental health conditions such as depression, anxiety, substance disorders, and suicidal ideation.[61-63,64] Men who have sex with men face social stigma in many settings, which can increase risk for HIV by limiting access to care.[65,66]. Thus, developing anti-stigma interventions and providing support that reduce homophobia and biphobia and encourage LGBT population rights, thereby reducing the amount of stigma, marginalization, and discrimination that MSM face in the first place would be very important in the future public health programmes. It is essential to change gradually social attitudes and reduce stigma and discrimination towards MSM populations. Health providers and MSM community organizations are valuable in providing social and emotional support to reduce stigma, alleviate mental disorder, and promote healthy behaviors. Besides, family members and friends from outside the MSM community also offer social resources after MSM disclose their sexual identity. Media campaigns against homophobia may

For peer review only

encourage broader acceptance of LGBT issues by portraying MSM in a non-discriminatory and normal manner.

There are some limitations to our study. First, these data were collected via self-report, and MSM might have concerns about their privacy and underreport some sensitive information in the rather homophobic and hostile environment of China. As such, this method may have resulted in an underestimation of the true prevalence of sensitive behaviors, such as unprotected sex, drug abuse, and sexual violence. Second, the causality of depressive symptoms and associated factors is not inferred due to the cross-sectional design, and the relevant information is subjected to recall bias, which may have an influence on the magnitude of associations with depression. Third, some important psychosocial factors, such as self-esteem, social support, and negative social relationships that might mediate or moderate the associations between depressive symptoms and related factors were not included in the research design. Additionally, stigma related to HIV and sexual stigma are important factors associated with depression[25] and were not examined. Fourth, different measures of depressive symptoms, such as the Self-Rating Depression Scale (SDS) and Beck Depression Inventory (BDI) may have provided results that differ from our study. Thus, longitudinal research with a larger sample size, employing more in-depth and complex measures of psychosocial and behavioral variables, and including such psychosocial factors as social support and social relationships, are needed to confirm our findings.

For peer review only - http://bmjopen.bmj.com/site/about/guidelines.xhtml

In response to our findings, mental health and substance abuse screening and counseling should be integrated into HIV prevention and treatment programs for the MSM population in China. Mental health issues are generally not given enough emphasis in the training of HIV/AIDS counselors in China, and we have had to provide supplementary training to staff from clinics and CDCs. More comprehensive intervention strategies that combine psychological, behavioral, and social aspects are needed to address the existing mental health issues, with special attention paid to suicidality and high-risk behaviors among MSM.

Author affiliations

[1]Center for Disease Control and Prevention of Zhejiang Province, Hangzhou, Zhejiang, China

[2]Department of Epidemiology & Biostatistics, School of Medicine, Ningbo University, Ningbo, Zhejiang, China

[3]Wenzhou Center for Disease Control and Prevention, Wenzhou, Zhejiang, China

[4] Lucheng Center for Disease Control and Prevention of, Wenzhou, Zhejiang, China.

Contributors SX, XP and QM were involved in the study design. HW, TJ, LH, SZ, DW, ZY, HZ and DZ performed the experiments. RL, XP and SX analysed the data. RL, XP and SX wrote the paper. XP, SX and QM critically revised the manuscript. All authors read and approved the final manuscript.

Acknowledgements We would like to thank Editage [www.editage.cn] for English language editing.

Funding Key Project on Social Development among S&T Major Project of Zhejiang Province, China (2013C03047-1).

Competing interests None declared.

Patient consent Obtained.

For peer review only - http://bmjopen.bmj.com/site/about/guidelines.xhtml

Ethics approval The study was approved by the ethical review board of Zhejiang Provincial Center for Disease Control and Prevention.

Data sharing statement No additional data are available.

References

1. Peirce RS, Frone MR, Russell M, *et al*. A longitudinal model of social contact, social support, depression, and alcohol use. ***Health Psychol*** 2000;19:28-38.
2. Anderson AM, Ross MW, Nyoni JE, *et al*. High prevalence of stigma-related abuse among a sample of men who have sex with men in Tanzania: implications for HIV prevention. ***AIDS Care*** 2015;27:63-70.
3. Risher K, Adams D, Sithole B, *et al*. Sexual stigma and discrimination as barriers to seeking appropriate healthcare among men who have sex with men in Swaziland. ***J Int AIDS Soc*** 2013;16:18715.
4. Cochran SD, Bandiera FC, Mays VM. Sexual orientation-related differences in tobacco use and secondhand smoke exposure among US adults aged 20 to 59 years: 2003-2010 National Health and Nutrition Examination Surveys. ***Am J Public Health*** 2013;103:1837-44.
5. Cochran SD, Mays VM. Lifetime prevalence of suicide symptoms and affective disorders among men reporting same-sex sexual partners: results from NHANES III. ***Am J Public Health*** 2000;90:573-8.
6. Farmer GW, Bucholz KK, Flick LH, *et al*. CVD risk among men participating in the National Health and Nutrition Examination Survey (NHANES) from 2001 to 2010: differences by sexual minority status. ***J Epidemiol Community Health*** 2013;67:772-8.
7. Guerim LD, de Carvalho HW, Lara DR. The relationship between temperament and sexual orientation. ***J Affect Disord*** 2015;175:379-84.
8. Mimiaga MJ, O'Cleirigh C, Biello KB, *et al*. The effect of psychosocial syndemic production on 4-year HIV incidence and risk behavior in a large cohort of sexually active men who have sex with men. ***J Acquir Immune Defic Syndr*** 2015;68:329-36.
9. Tucker A, Liht J, de Swardt G, *et al*. An exploration into the role of depression and self-efficacy on township men who have sex with men's ability to engage in safer sexual practices. ***AIDS Care*** 2013;25:1227-35.
10. Grov C, Rendina HJ, Ventuneac A, *et al*. HIV risk in group sexual encounters: an event-level analysis from a national online survey of MSM in the U.S. ***J Sex Med*** 2013;10:2285-94.
11. Baggaley RF, White RG, Boily MC. HIV transmission risk through anal intercourse: systematic review, meta-analysis and implications for HIV prevention. ***Int J Epidemiol*** 2010;39:1048-63.
12. Jin F, Jansson J, Law M, *et al*. Per-contact probability of HIV transmission in homosexual men in Sydney in the era of HAART. ***AIDS*** 2010;24:907-13.
13. Ministry of Health, People's Republic of China. 2012 China AIDS Response Progress Report. Beijing: Ministry of Health; 2012. http://unaids.org.cn/pics/20120614140133.pdf
14. Ma Q, Zeng S, Xia S, *et al*. Risky sexual networks and concentrated HIV epidemics among men who have sex with men in Wenzhou, China: a respondent-driven sampling study. ***BMC Public***

For peer review only

Health 2015;15:1246.

15. Mills TC, Paul J, Stall R, *et al*. Distress and depression in men who have sex with men: the Urban Men's Health Study. ***Am J Psychiatry*** 2004;161:278-85.

16. Liu J, Gao YH, Liang ZM, *et al*. Depressive symptoms and associated sexual behaviors among men who have sex with men in Foshan, Guangdong province [in Chinese]. ***Zhonghua Liu Xing Bing Xue Za Zhi*** 2012;33:483-7.

17. Wang Y, Zhang HB, Xu J, Zhang GG, Yang HW, Fan J. Survey of depression status among men having sex with men[in Chinese]. ***J Prev Med Inform*** 2010;26:597-600.

18. Feng Y, Wu Z, Detels R, *et al*. HIV/STD prevalence among men who have sex with men in Chengdu, China and associated risk factors for HIV infection. ***J Acquir Immune Defic Syndr*** 2010;53 Suppl 1:S74-80.

19. Zheng YJ, Xu J, Zhang HB. Anxiety, depression and high-risk behaviors among men who have sex with men [in Chinese]. ***Chinese Mental Health Journal*** 2005;19:699-701.

20. Wu J, Lu CY, Hao YT, *et al*. Psychosocial status of MSM population in Guangzhou city [in Chinese]. ***Chin J Public Health*** 2011;27:476-8.

21. Fang F, Zhang SP, Wang ZW, *et al*. Epidemiological survey of depression among residents in a community of Shanghai [in Chinese]. ***Chin J Behav Med & Brain Sci*** 2009;18:740-2.

22. Graham LF, Braithwaite K, Spikes P, et al. Exploring the mental health of black men who have sex with men. ***Community Ment Health J*** 2009;45:272-84.

23. De Santis JP, Colin JM, Provencio Vasquez E, *et al*. The relationship of depressive symptoms, self-esteem, and sexual behaviors in a predominantly Hispanic sample of men who have sex with men. ***Am J Mens Health*** 2008;2:314-21.

24. Salomon EA, Mimiaga MJ, Husnik MJ, *et al*. Depressive symptoms, utilization of mental health care, substance use and sexual risk among young men who have sex with men in EXPLORE: implications for age-specific interventions. ***AIDS Behav*** 2009;13:811-21.

25. Secor AM, Wahome E, Micheni M, *et al*. Depression, substance abuse and stigma among men who have sex with men in coastal Kenya. ***AIDS*** 2015;29 Suppl 3:S251-9.

26. Hirshfield S, Wolitski RJ, Chiasson MA, et al. Screening for depressive symptoms in an online sample of men who have sex with men. ***AIDS Care*** 2008;20:904-10.

27. Semple SJ, Stockman JK, Goodman-Meza D, *et al*. Correlates of Sexual Violence Among Men Who Have Sex With Men in Tijuana, Mexico. ***Arch Sex Behav*** 2016. [Epub ahead of print]

28. Strathdee SA, Hogg RS, Martindale SL, et al. Determinants of sexual risk-taking among young HIV-negative gay and bisexual men. J Acquir Immune Defic Syndr Hum Retrovirol 1998;19:61-6.

29. Reisner SL, Mimiaga MJ, Bland S, *et al*. Problematic alcohol use and HIV risk among Black men who have sex with men in Massachusetts. ***AIDS Care*** 2010;22:577-87.

30. Hernandez MT, Sanchez MA, Ayala L, *et al*. Methamphetamine and cocaine use among Mexican migrants in California: the California-Mexico Epidemiological Surveillance Pilot. ***AIDS Educ Prev*** 2009;21:34-44.

31. Blashill AJ, O'Cleirigh C, Mayer KH, *et al*. Body mass index, depression and sexual transmission risk behaviors among HIV-positive MSM. ***AIDS Behav*** 2012;16:2251-6.

For peer review only - http://bmjopen.bmj.com/site/about/guidelines.xhtml

32. Houston E, Sandfort T, Dolezal C, *et al*. Depressive symptoms among MSM who engage in bareback sex: does mood matter? ***AIDS Behav*** 2012;16:2209-2215.

33. Liu JX, Choi K. Experiences of social discrimination among men who have sex with men in Shanghai, China. ***AIDS Behav*** 2006; 10(4 Suppl):S25–33. [PubMed: 16715344]

34. Zhang B, Chu Q. MSM and HIV/AIDS in China. ***Cell Research*** 2005;15:858–64.

35. Feng Y, Wu Z, Detels R. Evolution of men who have sex with men community and experienced stigma among men who have sex with men in Chengdu, China. ***J Acquir Immune Defic Syndr*** 2010; 53(Suppl 1):S98–S103.

36. Steward WT, Miege P, Choi K. Charting a moral life: the influence of stigma and filial duties on marital decisions among Chinese men who have sex with men. ***PLoS One*** 2013; 8:e71778.

37. Zang C, Guida J, Sun Y, *et al*. Collectivism culture, HIV stigma and social network support in Anhui, China: a path analytic model. ***AIDS Patient Care STDS*** 2014;28(8):452–58.

38. King M, Semlyen J, Tai SS, *et al*. A systematic review of mental disorder, suicide, and deliberate self harm in lesbian, gay and bisexual people. ***Bmc Psychiatry*** 2008;8:70.

39. Paul JP, Catania J, Pollack L, Stall R. Understanding childhood sexual abuse as a predictor of sexual risk-taking among men who have sex with men: the Urban Men's Health Study. ***Child Abuse Negl*** 2001;25:557–84.

40. Ratner PA, Johnson JL, Shoveller JA, *et al*. Non-consensual sex experienced by men who have sex with men: prevalence and association with mental health. ***Patient Educ Couns*** 2003;49:67-74.

41. Deuba K, Ekström AM, Shrestha R, et al. Psychosocial health problems associated with increased HIV risk behavior among men who have sex with men in Nepal: a cross-sectional survey. PLoS One 2013;8:e58099.

42. O'Carroll PW, Berman AL, Maris RW, *et al*. Beyond the Tower of Babel: a nomenclature for suicidology. ***Suicide Life Threat Behav*** 1996; 26: 237–52.

43. Friedman MR, Stall R, Silvestre AJ, *et al*. Effects of syndemics on HIV viral load and medication adherence in the multicentre AIDS cohort study. ***AIDS*** 2015;29:1087-96.

44. Zhang J, Wu Z, Fang G, *et al*. Development of the Chinese age norms of CES-D in urban area [in Chinese]. ***Chinese Mental Health Journal*** 2010;24:139-43.

45. Zhang Y, Jia C, Fang Z, *et al*. Study on depression symptom and reliability and validity of CES - D used in rural residents in Shandong province [in Chinese]. ***Chin J Public Health*** 2008;24:473-5.

46. Heckathorn DD. Respondent-driven sampling: a new approach to the study of hidden populations. ***Soc Probl*** 1997;44:174–99.

47. Volz E, Heckathorn DD. Probability based estimation theory for respondent driven sampling. ***Journal of Official Statistics***. 2008;24:79-97.

48. Wejnert C. An Empirical Test of Respondent-Driven Sampling: Point Estimates, Variance, Degree Measures, and Out-of-Equilibrium Data. ***Sociological Methodology*** 2009;39:73-116.

49. Sandfort TG, Bakker F, Schellevis FG, *et al*. Sexual orientation and mental and physical health status: findings from a Dutch population survey. ***Am J Public Health*** 2006;96:1119-25.

50. Stoloff K, Joska JA, Feast D, *et al*. A description of common mental disorders in men who have sex with men (MSM) referred for assessment and intervention at an MSM clinic in Cape Town, South Africa. ***AIDS Behav*** 2013;17 Suppl 1:S77-81.

For peer review only - http://bmjopen.bmj.com/site/about/guidelines.xhtml

51. Tucker A, Liht J, de Swardt G, *et al*. Homophobic stigma, depression, self-efficacy and unprotected anal intercourse for peri-urban township men who have sex with men in Cape Town, South Africa: a cross-sectional association model. ***AIDS Care*** 2014;26:882-9.

52. Shiu CS, Chen YC, Tseng PC, *et al*. Curvilinear relationship between depression and unprotected sexual behaviors among men who have sex with men. ***J Sex Med*** 2014;11:2466-73.

53. Golub SA, Starks TJ, Payton G, et al. The critical role of intimacy in the sexual risk behaviors of gay and bisexual men. ***AIDS Behav*** 2012;16:626-32.

54. Craft SM, Smith SA, Serovich JM, *et al*. Need fulfillment in the sexual relationships of HIV-infected men who have sex with men. ***AIDS Educ Prev*** 2005;17:217-26.

55. Li HM, Peng RR, Li J, *et al*. HIV incidence among men who have sex with men in China: a meta-analysis of published studies. ***PLoS One*** 2011;6:e23431.

56. Li R, Cai Y, Wang Y, *et al*. Psychosocial Syndemic Associated With Increased Suicidal Ideation Among Men Who Have Sex With Men in Shanghai, China. ***Health Psychol*** 2016;35:148-56.

57. Roura M, Urassa M, Busza J, *et al*. Scaling up stigma? The effects of antiretroviral roll-out on stigma and HIV testing. Early evidence from rural Tanzania. ***Sex Transm Infect*** 2009;85:308-12.

58. Sivasubramanian M, Mimiaga MJ, Mayer KH, *et al*. Suicidality, clinical depression, and anxiety disorders are highly prevalent in men who have sex with men in Mumbai, India: findings from a community-recruited sample. ***Psychol Health Med*** 2011;16:450-62.

59. Maslowsky J, Schulenberg JE, O'Malley PM, *et al*. Depressive symptoms, conduct problems, and risk for polysubstance use among adolescents: Results from US national surveys. ***Ment Health Subst Use*** 2013;7:157-69.

60. Schneider B, Kolves K, Blettner M, *et al*. Substance use disorders as risk factors for suicide in an Eastern and a Central European city (Tallinn and Frankfurt/Main). ***Psychiatry Res*** 2009;165:263-72.

61. Logie CH, Newman PA, Chakrapani V, *et al*. Adapting the minority stress model: associations between gender nonconformity stigma, HIV-related stigma and depression among men who have sex with men in South India. ***Soc Sci Med*** 2012;74:1261–8.,

62. Meyer IH. Prejudice, social stress, and mental health in lesbian, gay, and bisexual populations: conceptual issues and research evidence. ***Psychol Bull*** 2003;129:674–97.

63. Stahlman S, Grosso A, Ketende S, *et al*. Depression and Social Stigma Among MSM in Lesotho : Implications for HIV and Sexually Transmitted Infection Prevention. ***AIDS Behav*** 2015;19:1460–9.

64. Williamson IR. Internalized homophobia and health issues affecting lesbians and gay men. ***Health Educ Res*** 2000;15:97–107.

65. Stahlman S, Bechtold K, Sweitzer S, *et al*. Sexual identity stigma and social support among men who have sex with men in Lesotho: a qualitative analysis. ***Reprod Health Matters*** 2015;23:127-35.

66. Zhao Y, Zhang L, Zhang H, ***et al***. HIV Testing and Preventive Services Accessibility Among Men Who Have Sex With Men at High Risk of HIV Infection in Beijing, China. ***Medicine (Baltimore)***. 2015 Feb;94(6):e534.

For peer review only - http://bmjopen.bmj.com/site/about/guidelines.xhtml

Table 1. Sociodemographic characteristics and their associations with depressive symptoms among MSM in Wenzhou, China (N = 454)

Variables	Total n(%)	Major depressive symptoms n(%)	OR (95%CI)	*P* value
**Age(years)** (median = 33; IQR, 26–41)				
≤25	101(22.2)	39(38.6)	1.70(1.01-2.84)	0.044
26–35	168(37.0)	68(40.5)	1.84(1.17-2.87)	0.008
36 and older	185(40.7)	50(27.0)	1.00	
**Marital status**				
Never married	253(55.7)	97(38.3)	1.00	
Married/Divorced/Widowed	201(44.3)	60(29.9)	0.68(0.46-0.99)	0.049
**Education level**				
Junior school or lower	272(59.9)	87(32.0)	1.00	
Senior school	121(26.7)	50(41.3)	1.50(0.96-2.33)	0.074
College or higher	61(13.4)	20(32.8)	1.04(0.57-1.88)	0.904
**Monthly income (RMB)**				
<3000	194(42.7)	62(32.0)	1.00	
3000 and above	260(57.3)	95(36.5)	1.23(0.83-1.82)	0.310
**Duration of residence in Wenzhou (years)**				
≤3	220(48.6)	79(35.9)	1.00	
>3	233(51.4)	77(33.0)	0.88(0.60-1.30)	0.522
**Insurance**				
Insured	140(30.8)	54(38.6)	1.00	
Uninsured	314(69.2)	103(32.8)	0.78(0.51-1.18)	0.233
**Sexual orientation**				
Homosexual	184(40.5)	71(38.6)	1.34(0.91-1.99)	0.139

For peer review only

Heterosexual or bisexual or indeterminate	270(59.5)	86(31.9)	1.00	
**Places to seek for sexual partners**				
Venues	261(59.9)	85(32.6)	1.00	
Internet	175(40.1)	66(37.7)	1.25(0.84-1.87)	0.269
**Number of MSM which a participant knew in his circle of MSM friends locally** (median = 7; IQR, 3–15)				
>7	220(48.5)	74(33.6)	0.92(0.63-1.36)	0.681
≤7	234(51.5)	83(35.5)	1.00	

MSM = men who have sex with men, OR = odds ratio, CI = confidence interval, IQR = interquartile range.

Table 2. Sexual behavior and their associations with depressive symptoms among MSM in Wenzhou, China

(N = 454)

**Variables**	**Total n(%)**	**Major depressive symptoms n(%)**	**OR (95%CI)**	***P* value**
**Age of sexual debut with a man** (median = 24; IQR, 20–30)				
≤22	197(43.4)	70(35.5)	1.08(0.73-1.59)	0.709
>22	257(56.6)	87(33.9)	1.00	
**Anal intercourse with men in the past 6 months**				
Yes	386(85.0)	139(36.0)	1.56(0.88-2.79)	0.129
No	68(15.0)	18(26.5)	1.00	
**Number of anal intercourse male partners in the past 6 months** (median = 2; IQR, 1–3)				
≤2	307(67.6)	98(31.9)	1.00	

For peer review only

For peer review only - http://bmjopen.bmj.com/site/about/guidelines.xhtml

>2	147(32.4)	59(40.1)	1.43(0.95-2.15)	0.086
**Consistent condom use during anal sex in the past 6 months**				
Yes	174(45.1)	46(26.4)	1.00	
No	212(54.9)	93(43.9)	2.18(1.41-3.35)	<0.001
**Oral sex with men in the past 6 months**				
Yes	348(76.7)	124(35.6)	1.23(0.77-1.95)	0.394
No	106(23.3)	33(31.1)	1.00	
**Number of oral male sexual partners in the past 6 months**				
≤2	309(68.1)	97(31.4)	1.00	
>2	145(31.9)	60(41.4)	1.54(1.03-2.32)	0.038
**Consistent condom use during oral sex in the past 6 months**				
Yes	69(19.8)	24(34.8)	1.00	
No	279(80.2)	100(35.8)	1.05(0.60-1.82)	0.869
**Sex with women in the past**				
Yes	348(76.7)	115(33.0)	0.75(0.48-1.18)	0.213
No	106(23.3)	42(39.6)	1.00	
**Age of sexual debut with a woman**				
≤22	209(60.1)	71(34.0)	1.11(0.70-1.76)	0.653
>22	139(39.9)	44(31.7)	1.00	
**Sex with women in the past 6 months**				
Yes	167(36.8)	59(35.3)	1.05(0.71-1.57)	0.798
No	287(63.2)	98(34.1)	1.00	
**Number of female sex partners in the past 6 months**				
<2	407(90.0)	137(33.7)	1.00	
≥2	45(10.0)	19(42.2)	1.44(0.77-2.69)	0.254
**Consistent condom use during vaginal sex with women in the past 6 months**				
Yes	53(31.7)	21(39.6)	1.00	

For peer review only - http://bmjopen.bmj.com/site/about/guidelines.xhtml

No	114(68.3)	38(33.3)	0.76(0.39-1.50)	0.429

MSM = men who have sex with men, OR = odds ratio, CI = confidence interval, IQR = interquartile range.

Table 3. Substance use, psychosocial factors and their associations with depressive symptoms among MSM in Wenzhou, China (N = 454)

Variables	Total n(%)	Major Depressive symptoms n(%)	Odds Ratio (95%CI)	*P* value
**Smoking**				
Yes	284(62.6)	98(34.5)	0.99(0.67-1.48)	0.966
No	170(37.4)	59(34.7)	1.00	
**Drinking alcohol**				
Yes	188(41.4)	64(34.0)	0.96(0.65-1.42)	0.839
No	266(58.6)	93(35.0)	1.00	
**Being drunk once in the past year**				
Yes	87(46.3)	39(44.8)	2.47(1.33-4.59)	0.004
No	101(53.7)	25(24.8)	1.00	
**Wanting or needing to reduce drinking in the past year**				
Yes	48(55.2)	26(54.2)	2.36(0.99-5.67)	0.054
No	39(44.8)	13(33.3)	1.00	
**Drug use**				
Yes	17(3.7)	8(47.1)	1.72(0.65-4.55)	0.275
No	437(96.3)	149(34.1)	1.00	

For peer review only - http://bmjopen.bmj.com/site/about/guidelines.xhtml

For peer review only

**Suicidal ideation**				
Yes	73(16.1)	50(68.5)	5.57(3.24-9.57)	<0.001
No	381(83.9)	107(28.1)	1.00	
**Adult sexual violence from same-sex partners**				
Yes	36(7.9)	23(63.9)	3.75(1.84-7.63)	<0.001
No	418(92.1)	134(32.1)	1.00	
**Childhood sexual abuse**				
Yes	23(5.1)	14(60.9)	3.13(1.32-7.41)	0.009
No	431(94.9)	143(33.2)	1.00	

MSM = men who have sex with men, OR = odds ratio, CI = confidence interval.

Table 4. RDS-weighted multivariable analyses of factors associated with depressive symptoms among MSM in Wenzhou, China (N = 454)

Variable	AOR(95%CI)	*P* value	RDS-weighted AOR(95%CI)	*P* value
**Age(years)**				
≤25	1.26(0.62-2.54)	0.526	1.17(0.56-2.43)	0.681
26–35	1.74(1.01-3.01)	0.048	2.43(1.35-4.39)	0.003
36 and older	1		1	
**Marital status**				
Never married	1		1	
Married/Divorced/Widowed	0.73(0.43-1.24)	0.246	0.64(0.36-1.12)	0.119
**Education level**				
Junior school or lower	1		1	
Senior school	1.55(0.92-2.62)	0.103	2.01(1.16-3.49)	0.014
College or higher	1.08(0.53-2.20)	0.838	0.92(0.45-1.88)	0.825
**Monthly income (RMB)**				
<3000	1		1	
3000 and above	0.92(0.58-1.46)	0.714	0.65(0.40-1.03)	0.068
**Sexual orientation**				
Homosexual	1.05(0.67-1.66)	0.823	0.85(0.52-1.37)	0.499
Heterosexual or bisexual or	1		1	

For peer review only

indeterminate				
**Consistent condom use during anal sex with men in the past 6 with months**				
Yes	1		1	
No	2.13(1.32-3.45)	0.002	3.62(2.15-6.13)	<0.001
**Number of oral male sexual partners in the past 6 months**				
≤2	1		1	
>2	1.44(0.91-2.27)	0.12	1.75(1.09-2.80)	0.02
**Being drunk once in the past year**				
Yes	2.07(1.00-4.26)	0.049	2.89(1.36-6.14)	0.006
No	1		1	
**Suicidal ideation**				
No	1		1	
Yes	4.54(2.50-8.26)	<0.001	5.09(2.59-9.97)	<0.001
**Adult sexual violence from male sexual partners**				
No	1		1	
Yes	2.25(1.00-5.07)	0.051	2.57(1.03-6.73)	0.031
**Childhood sexual abuse**				
Yes	1.84(0.63-5.36)	0.263	1.89(0.34-10.57)	0.467
No	1		1	

MSM = men who have sex with men, AOR = adjusted odds ratio, CI = confidence interval; RDS=respondent-driving sampling.

563

For peer review only - http://bmjopen.bmj.com/site/about/guidelines.xhtml

STROBE 2007 (v4) Statement—Checklist of items that should be included in reports of *cross-sectional studies*

Section/Topic	Item #	Recommendation	Reported on page #
**Title and abstract**	1	(*a*) Indicate the study's design with a commonly used term in the title or the abstract	1-3
		(*b*) Provide in the abstract an informative and balanced summary of what was done and what was found	2-3
**Introduction**			
Background/rationale	2	Explain the scientific background and rationale for the investigation being reported	4-6
Objectives	3	State specific objectives, including any prespecified hypotheses	5-6
**Methods**			
Study design	4	Present key elements of study design early in the paper	6-7
Setting	5	Describe the setting, locations, and relevant dates, including periods of recruitment, exposure, follow-up, and data collection	6-7
Participants	6	(*a*) Give the eligibility criteria, and the sources and methods of selection of participants	6
Variables	7	Clearly define all outcomes, exposures, predictors, potential confounders, and effect modifiers. Give diagnostic criteria, if applicable	7-9
Data sources/ measurement	8*	For each variable of interest, give sources of data and details of methods of assessment (measurement). Describe comparability of assessment methods if there is more than one group	7-9
Bias	9	Describe any efforts to address potential sources of bias	*10*
Study size	10	Explain how the study size was arrived at	7
Quantitative variables	11	Explain how quantitative variables were handled in the analyses. If applicable, describe which groupings were chosen and why	10
Statistical methods	12	(*a*) Describe all statistical methods, including those used to control for confounding	10
		(*b*) Describe any methods used to examine subgroups and interactions	
		(*c*) Explain how missing data were addressed	
		(*d*) If applicable, describe analytical methods taking account of sampling strategy	10
		(*e*) Describe any sensitivity analyses	
**Results**			

For peer review only

Participants	13*	(a) Report numbers of individuals at each stage of study—eg numbers potentially eligible, examined for eligibility, confirmed eligible, included in the study, completing follow-up, and analysed	11-13
		(b) Give reasons for non-participation at each stage	
		(c) Consider use of a flow diagram	
Descriptive data	14*	(a) Give characteristics of study participants (eg demographic, clinical, social) and information on exposures and potential confounders	11-13
		(b) Indicate number of participants with missing data for each variable of interest	
Outcome data	15*	Report numbers of outcome events or summary measures	11-13
Main results	16	(*a*) Give unadjusted estimates and, if applicable, confounder-adjusted estimates and their precision (eg, 95% confidence interval). Make clear which confounders were adjusted for and why they were included	11-12
		(*b*) Report category boundaries when continuous variables were categorized	12-13
		(*c*) If relevant, consider translating estimates of relative risk into absolute risk for a meaningful time period	
Other analyses	17	Report other analyses done—eg analyses of subgroups and interactions, and sensitivity analyses	
**Discussion**			
Key results	18	Summarise key results with reference to study objectives	13
Limitations	19	Discuss limitations of the study, taking into account sources of potential bias or imprecision. Discuss both direction and magnitude of any potential bias	17
Interpretation	20	Give a cautious overall interpretation of results considering objectives, limitations, multiplicity of analyses, results from similar studies, and other relevant evidence	13-17
Generalisability	21	Discuss the generalisability (external validity) of the study results	17
**Other information**			
Funding	22	Give the source of funding and the role of the funders for the present study and, if applicable, for the original study on which the present article is based	18

*Give information separately for cases and controls in case-control studies and, if applicable, for exposed and unexposed groups in cohort and cross-sectional studies.

Note: An Explanation and Elaboration article discusses each checklist item and gives methodological background and published examples of transparent reporting. The STROBE checklist is best used in conjunction with this article (freely available on the Web sites of PLoS Medicine at http://www.plosmedicine.org/, Annals of Internal Medicine at http://www.annals.org/, and Epidemiology at http://www.epidem.com/). Information on the STROBE Initiative is available at www.strobe-statement.org.

For peer review only

Sexual risk behavior, sexual victimization, substance use, and other factors related to depression in men who have sex with men in Wenzhou, China: a cross-sectional study

Journal:	*BMJ Open*
Manuscript ID	bmjopen-2016-013512.R2
Article Type:	Research
Date Submitted by the Author:	20-Apr-2017
Complete List of Authors:	Pan, Xiaohong; Center for Disease Control and Prevention of Zhejiang Province<br>Li, Runhua; Ningbo University, Medical school<br>Ma, Qiaoqin; Center for Disease Control and Prevention of Zhejiang Province, ;<br>Wang, Hui; Center for Disease Control and Prevention of Zhejiang Province,<br>Jiang, Tingting; Center for Disease Control and Prevention of Zhejiang Province,<br>He, Lin; Center for Disease Control and Prevention of Zhejiang Province,<br>Zeng, Shidian; Wenzhou Center for Disease Control and Prevention,<br>Wang, Dayong; Wenzhou Center for Disease Control and Prevention,<br>Ye, Zhenmiao; Wenzhou Center for Disease Control and Prevention,<br>Zhu, Haishen; Center for Disease Control and Prevention of Lucheng District, Wenzhou<br>Zhao, Dongshe; Lucheng Center for Disease Control and Prevention<br>Xia, Shichang; Center for Disease Control and Prevention of Zhejiang Province,
**Primary Subject Heading**:	Public health
Secondary Subject Heading:	HIV/AIDS, Mental health
Keywords:	EPIDEMIOLOGY, HIV & AIDS < INFECTIOUS DISEASES, Public health < INFECTIOUS DISEASES, MENTAL HEALTH

For peer review only - http://bmjopen.bmj.com/site/about/guidelines.xhtml

Sexual risk behavior, sexual victimization, substance use, and other factors related to depression in men who have sex with men in Wenzhou, China: a cross-sectional study

#Xiaohong Pan[1], #Runhua Li[1,2], Qiaoqin Ma[1], Hui Wang[1], Tingting Jiang[1], Lin He[1], Shidian Zeng[3], Dayong Wang[3], Zhenmiao Ye[3], Haishen Zhu[4], Dongshe Zhao[4], *Shichang Xia[1]

[1]Center for Disease Control and Prevention of Zhejiang Province, Hangzhou, Zhejiang, China

[2]Department of Epidemiology & Biostatistics, School of Medicine, Ningbo University, Ningbo, Zhejiang, China

[3]Wenzhou Center for Disease Control and Prevention, Wenzhou, Zhejiang, China

[4] Lucheng Center for Disease Control and Prevention, Wenzhou, Zhejiang, China.

*Corresponding author: Shichang Xia

Email: shchxia@cdc.zj.cn

#Xiaohong Pan, and Runhua Li contributed equally to this work.

For peer review only - http://bmjopen.bmj.com/site/about/guidelines.xhtml

Abstract

Objectives: Men who have sex with men (MSM) are a marginalized population at high risk for a variety of mental health problems that may mutually facilitate HIV transmission. We assessed factors associated with depressive symptoms among MSM, which can provide some guidance for future mental health interventions with the MSM population for prevention of HIV infection and transmission. **Methods:** A cross-sectional study was conducted in Wenzhou City, China using respondent-driven sampling between December 2013 and June 2014. A face-to-face questionnaire survey was employed to collect information about mental and psychosocial health conditions and sexual behavior characteristics among MSM. Bivariate and multivariable logistic regression analyses were used to identify factors associated with major depressive symptoms. **Results:** Of the 454 participants in the study, prevalence of major depressive symptoms was 34.6%. During the past 6 months, 85% had anal sex with men, and rate of consistent condom use during anal intercourse was 45.1%. Of the respondents, 16.1% reported suicidal ideation, 62.6% had a history of smoking, and 41.4% had a history of drinking alcohol, of which 46.3% reported that they were once drunk. Drug use was reported in 3.7% of the sample. Adult sexual violence from same-sex partners was 7.9%, and 5.1% reported childhood sexual abuse. A RDS-weighted multivariable analysis showed that major depressive symptoms among MSM were associated with inconsistent condom use during anal sex with men; multiple oral male sexual partners； suicidal ideation; adult sexual violence from male partners; and being once drunk in the past year. **Conclusion:** We found high

For peer review only - http://bmjopen.bmj.com/site/about/guidelines.xhtml

levels of depressive symptoms, unprotected sex, and suicidal ideation among MSM. Comprehensive intervention strategies that combine psychological, behavioral, and social aspects are needed to address the mental health issues of MSM, with special attention given to suicidality and high-risk behaviors in mental health and HIV prevention interventions.

Strengths and limitations of this study

1. The present study obtained the prevalence of major depressive symptoms, sexual risk behaviors, sexual victimization, suicidal ideation, substance use and elucidated some related syndemic factors to depressive symptoms among MSM in Wenzhou with high HIV prevalence.

2. Our study can provide some guidance for mental health and HIV prevention interventions for the MSM population.

3. It is unavoidable that MSM might underreport some sensitive issues such as unprotected sex, drug abuse, and sexual violence covered in the questionnaire in the rather homophobic and hostile environment of China.

4. The causality of depressive symptoms and associated risk factors is not inferred due to the cross-sectional design, and the relevant information is subjected to recall bias, which may have an influence on the magnitude of associations with depression.

For peer review only - http://bmjopen.bmj.com/site/about/guidelines.xhtml

Introduction

Men who have sex with men (MSM) are a marginalized population facing many challenges including stigma, abuse, social and legal discrimination, and lack of social support,[1-3] which may result in high risk for a variety of mental and psychosocial health problems, such as depression, suicidality, and drug and alcohol abuse.[4-7] A large prospective cohort study showed that syndemics of depressive symptoms, heavy alcohol use and drug use, and childhood sexual abuse (CSA) increased HIV risk and HIV-related risk behaviors.[8] Depression and substance abuse among MSM have been linked to condomless anal intercourse (CAI),[9,10] which was the predominant factor of HIV acquisition and transmission.[11,12] These interactive factors have resulted in high HIV prevalence and incidence among MSM. It is estimated that MSM accounted for 29.4 % of the new HIV infections in China in 2011.[13] In Wenzhou City of China, the prevalence of HIV was estimated to be 22.8% (95% confidence interval [CI], 16.9 to 28.5%) in a previous publication.[14]

A survey conducted in four large American cities showed the prevalence of depressive symptoms among MSM was 29.2%, nearly three times that of the general population.[15] In China, the prevalence of depressive symptoms among MSM has varied from 34.8% to 63.9%,[16-20] which was much higher than 5.3% among the general population.[21] Previous studies showed that factors associated with depression among MSM included race,[22] lower educational level,[23] younger age,[24] and marriage to a

For peer review only - http://bmjopen.bmj.com/site/about/guidelines.xhtml

woman.[25,26] Psychological factors such as sexual victimization,[27,28] substance abuse,[29,30] and HIV or sexual stigma[25] were associated with depressive symptoms among MSM. Some studies showed that engaging in high-risk sexual behaviors was associated with depressive symptoms among the MSM population.[31,32] In China, homosexual behavior has been subject to prejudice because it continues to be seen by many people as a rejection of China's cultural tradition to marry and have children.[33,34] The marriage between two men was also not admitted in Chinese laws. Chinese MSM have suffered much social stigma and discrimination from the whole society due to their sexual orientation.[33,35,36] Prior research suggests that the pressures tied to maintaining their needs of homosexual behavior and culturally normative social duties are associated with stress and internalized feelings of blame and low self-worth among Chinese MSM.[36,37] Minority stress theory has been proposed to explain the well-documented prevalence of various mental health problems, such as depression and anxiety symptoms, and suicidal thoughts among MSM.[38]

Few studies have examined the associations between depressive symptoms among MSM and sexual risk behaviors, sexual victimization, and substance use, which is very important to guide mental health and HIV interventions among MSM in China. These previous studies were conducted to examine several independent factors associated with depression and did not comprehensively consider integrating sociodemographic characteristics, high-risk sexual behaviors, psychosocial factors and substance use into their analyses.

For peer review only

For peer review only - http://bmjopen.bmj.com/site/about/guidelines.xhtml

Some studies conducted in the US and in European countries examined depression and associated factors among MSM, while such research in China has been limited and lacks comprehensiveness. In the present study, our endeavor was to ascertain the prevalence of depression in Wenzhou City, China and related syndemic factors, including sexual risk behaviors, suicidality, sexual victimization, and substance use. Such knowledge would provide guidance for mental health and HIV prevention interventions for the MSM population.

Materials and methods

Study population and sampling

This cross-sectional study was conducted in Wenzhou City, China between December 2013 and June 2014. This baseline survey research was conducted for the purposes of controlling HIV/AIDS new infections among the MSM population in Zhejiang Province, China. The study procedures and sampling strategies are described in detail elsewhere.[14] Briefly, participants were recruited using a respondent−driving sampling (RDS) method. Participants who were at least 16 years old, had resided in Wenzhou at least 3 months, self-reported having had anal sex and/or oral sex in the past 12 months, and were willing to participate in the survey met inclusion criteria in this study. First, we selected 5 initial active "seed" participants meeting inclusion criteria from the local MSM population. After completing the field investigation and relevant testing, "seed" participants were provided with 3 recruitment coupons, and through the

For peer review only - http://bmjopen.bmj.com/site/about/guidelines.xhtml

incentive measures the "seed" participants then referred a maximum of 3 respondents from their social network to participate in the survey. These respondents continued to refer a maximum of 3 peers to the study and continued to recruit MSM participants until equilibrium was reached. The sample composition was considered to have reached equilibrium when their socio-demographic characteristics were stable, and would not change much for further recruitment of participants (the key socio-demographic characteristics such as age, marital status, education level, monthly income and sexual orientation changed <2% in a subsequent wave recruitment of peers compared with the previous one), irrespective of the characteristics of the initial seeds. All participants received remuneration in the amount of RMB 100 (approximately US$ 16) for participation and another RMB 100 as an incentive to refer peers to participate in the study. Finally, 5 seeds recruited 30, 61, 81, 103, and 174 MSM, respectively, with a total sample of 454 MSM by 14 waves of recruitment. All eligible participants were interviewed by trained staff from the Center for Disease Control and Prevention of Lucheng District, Wenzhou, who also collected the questionnaire about sociodemographics, sexual risk behaviors, psychosocial conditions, HIV-related cognitions, and HIV testing history of participants.

Measures

Mental and psychosocial health conditions

Depression. Depressive symptoms were assessed using the Center for Epidemiological Studies Depression Scale (CES-D). The CES-D consists of 20 items

For peer review only - http://bmjopen.bmj.com/site/about/guidelines.xhtml

designed to assess the experience of depression. Respondents indicate the extent to which each item statement describes their experience during the past week. Response options include the following: “rarely or none of the time (less than 1 day),” “some or a little of the time (1–2 days),” “occasionally or a moderate amount of the time (3–4 days),” and “most or all of the time (5–7 days).” Responses are on a 4-point Likert sale and scored 0–3. Level of depression was categorized as follows: major depressive symptoms (CES-D score of 20 or higher), moderate depressive symptoms (CES-D score of 16–19), and no depressive symptoms (CES-D score of 15 or lower). Thus, higher scores indicate higher levels of depressive symptoms. A total scale score of 20 or higher was used to define major depressive symptoms as the main analysis variable in our study.

Sexual victimization. Sexual victimization included childhood sexual abuse and adult sexual violence from same-sex partners. We defined childhood sexual abuse as the experience of being “forced or frightened by someone into doing something sexually” with a partner more than 10 years older than the respondent when the respondent was aged 15 years or younger.[39] Those respondents who reported that they were first forced or coerced to have sex against their will by men when 16 years of age or older were classified as having experienced adult sexual violence. These respondents did not report experiencing childhood sexual abuse (i.e. when under the age of 16 years).[40,41]

Suicidal ideation. Participants were asked: “during the past year, have you ever had thoughts of taking your own life?” The definition of suicidal ideation referred to previous research.[42]

For peer review only - http://bmjopen.bmj.com/site/about/guidelines.xhtml

Use of substances

Substance use included smoking cigarettes, alcohol consumption, and use of drugs. Cigarette smoking refers to smoking one or more cigarettes every day for more than a year or smoking more than 300 cigarettes in 3 months or less. Alcohol consumption refers to ethanol intake of 100 grams per week such as 250 grams of 40% volume of wine consumption or 1 kilogram of 10% volume of red wine intake. According to the definition, participants were asked whether they had a history of smoking and drinking alcohol, whether they had been drunk in the past year, and whether they wanted or needed to reduce drinking in the past year. Drug use were defined as using at least two kinds of the following drugs at least monthly during the past 12 months: sedatives, morphine, mescalinum, LSD, opiates, cocaine, marijuana, heroin, ecstasy, other street or club drugs.[43]

Sexual risk behaviors

Sexual risk behaviors included 6 items: 1) number of anal intercourse male partners in the past 6 months, 2) consistent condom use during anal sex with men, 3) number of oral sex with male partners in the past 6 months, 4) consistent condom use during oral sex with men, 5) number of female sexual partners, and 6) consistent condom use during vaginal intercourse with women.

Translation, validation and reliability

Measures were independently translated from English to Chinese by two staff members fluent in both languages. The surveys were then back-translated into English to

For peer review only

For peer review only - http://bmjopen.bmj.com/site/about/guidelines.xhtml

ensure that they retained their original meaning and good validation of the translation. The Cronbach's alpha coefficient of the CES-D in a large population from 39 cities in China was 0.90.[44] The Cronbach' alpha coefficient of the CES-D was 0.88 for this sample. The scale is fit for Chinese population with good validation and reliability. [44,45]

Statistical analysis

Data were entered into EpiData version 3.1 (http://www.epidata.dk/) via double entry. After data cleaning and verification, the data were processed using PASW Statistics 18.0 (SPSS Inc., Chicago, IL, USA) for statistical analysis. For descriptive analyses, the mean, standard deviation (SD), median, and interquartile range (IQR) were computed for continuous variables and frequencies for categorical variables. Multicollinearity was diagnosed by examining the variance inflation factors (VIF). Variables significantly associated with major depressive symptoms at the level of $P < .05$ in the univariate logistic analyses were included in the multivariable logistic regression model, controlling for demographic variables (age, marital status, education level, monthly income, and sexual orientation) and calculating adjusted odds ratios (AOR) and 95% confidence intervals (CI). Then, we used the Respondent-Driven Sampling Analysis Tool (RDSAT) version 7.1 (http://www.respondentdrivensampling.org/) to produce individualized weights for adjusting data of different participants with different network sizes.[46-48] The individualized weights were imported into PASW Statistics 18.0 to conduct weighted multivariable logistic regression analyses. Statistical significance was defined as $P < .05$.

Ethics statement

For peer review only

The study was approved by the ethical review board of Zhejiang Provincial Center for Disease Control and Prevention. No harm was given to the participants by the study and we protected confidentiality of the participants. A written informed consent was signed by all participants during the survey.

Results

Sample characteristics

A total of 454 MSM participated in the study, and the characteristics of the sample are provided in Table 1. Of the 454 participants, 55.7% (253/454) were never married and 51.4% (233/453) resided in Wenzhou City for more than three years. The age of the respondents ranged from 17 to 76 years (median = 33; IQR, 26–41). Regarding education level, 86.6% (393/454) had an education of senior school or lower. Roughly sixty percent (57.3%) of the participants had an income of not less than 3000 RMB monthly, 30.8% (140/454) had health insurance, and 40.5% reported their sexual orientation as homosexual or gay(Table 1).

Depressive symptoms, sexual risk behaviors, suicidal ideation, substance use, and sexual victimization

The median CES-D scale score was 16 (IQR, 12–22; Range, 0–44). The prevalence of major depressive symptoms (CES-D score ⩾20) was 34.6% (157/454), while 53.5% (243/454) of the participants had moderate to major depressive symptoms (CES-D score ⩾16) and 46.5% (211/454) had no depressive symptoms (CES-D score ⩽15). Of the 454 participants, 85.0% (386/454) had anal sex with men during the past 6 months and

For peer review only - http://bmjopen.bmj.com/site/about/guidelines.xhtml

the rate of consistent condom use during anal intercourse was 45.1% (174/386), and 76.7% (348/454) had oral sex with men during the past 6 months and the percentage of consistent condom use during oral sex was 19.8% (69/348). Further, 32.4% reported having anal sex with more than 2 same-sex partners during the past 6 months and 31.9% engaged in oral sex during the past 6 months. Suicidal ideation was reported by 16.1% (73/454) of all respondents. History of smoking was reported in 62.6% (284/454) of the participants, and 41.4% (188/454) had a history of drinking alcohol of which 46.3% (87/188) indicated they were once drunk. Only 3.7% (17/454) had used drugs. Regarding sexual victimization, 7.9% (36/454) had suffered adult sexual violence from same-sex partners and 5.1% (23/454) reported sexual abuse in childhood. (Table 2–3).

Factors associated with depressive symptoms

Results of univariate logistic regression analyses are shown in Tables 1–4. Young age, never-been-married, no consistent condom use during anal sex in the past 6 months, number of oral sex male partners in the past 6 months, being once drunk in the past year, suicidal ideation, suffering violence from same-sex partners, and experiencing sexual abuse in childhood were associated with depressive symptoms.

Results of the RDS-weighted multivariable analyses are shown in Table 4. Major depressive symptoms were associated with inconsistent condom use during anal sex in the past 6 months (AOR = 3.62; 95%CI, 2.15-6.13; $P < .001$); more than two oral sexual male partners in the past 6 months (AOR = 1.75; 95%CI, 1.09-2.80; $P = .020$); suicidal ideation (AOR = 5.09; 95%CI, 2.59-9.97; $P < .001$); suffering adult violence from

For peer review only - http://bmjopen.bmj.com/site/about/guidelines.xhtml

For peer review only

same-sex partners (AOR = 2.57; 95%CI, 1.03-6.73; P = 0.031); being once drunk in the past year (AOR = 2.89; 95%CI, 1.36-6.14; P = .006).

Discussion

This is the first study to explore a comprehensive range of factors for their association with depressive symptoms among MSM in China. Specifically, we investigated risky sexual behaviors, substance use, and psychosocial factors including suicidal thoughts, and sexual victimization for their relationship to depressive symptoms. We found high rates of depressive symptoms, unprotected sex, and suicidal ideation in our sample. Additionally, we found that suffering adult sexual violence from same-sex partners was associated with depressive symptoms.

Our study showed a high rate of depressive symptoms in MSM; higher than the reported prevalence rate of depressive symptoms among the general population in China of 5.3%.[21] A similar increased risk of depressive symptoms has been demonstrated among MSM in the USA, Netherlands, Kenya, and South Africa.[15,25,49-51] Due to the prevalence of depressive symptoms among the MSM population greatly exceeding that of the general population, we should lay emphasis on psychosocial assessment of and interventions for MSM.

It is noteworthy to have an association between depressive symptoms and CAI (i.e., inconsistent condom use during anal intercourse) in our study, in line with the study conducted by Houston et al.[32] It is possible that MSM with mild to moderate depressive

For peer review only - http://bmjopen.bmj.com/site/about/guidelines.xhtml

symptoms address their low mood by having unprotected sex, which would increase the sense of intimacy and pleasure.[52-54] Studies have shown that CAI among MSM was the predominant factor in HIV acquisition and transmission.[11,12] Of particular concern were the rising incidence and high prevalence of HIV and lower rate of condom use when engaging in anal sex among MSM in China, specially in Wenzhou.[14,55] Therefore, mental health interventions that focus on reducing high risk sexual behaviors and improving mental health among MSM may be effective strategies for preventing HIV infection and transmission. Additionally, future HIV prevention programs should consider incorporating mental health screening and intervention strategies to improve their effectiveness in promoting sexual health.

In our study, we found a high rate of suicidal ideation and a strong association between suicidal ideation and depressive symptoms. Li et al., in his recent study conducted in Shanghai, China, argued that not feeling a sense of belonging and having little social support may explain the high rates of suicidal ideation in MSM,[-56] and the lack of a sense of belonging and social support may cause mental health issues such as depressive symptoms. A longitudinal study revealed that lesbian, gay, bisexual, and transgender (LGBT) youth with a history of suicide attempt were found to have higher levels of depressive and anxious symptoms.[57] Conversely, MSM with higher levels of self-esteem and greater satisfaction with social support were less likely to have major depression and suicidal ideation.[58] Given the positive association between major depression and suicidal ideation, a sense of belonging and social support may serve as

For peer review only - http://bmjopen.bmj.com/site/about/guidelines.xhtml

protective factors as well as mediate the relationship between depression and suicidality. Given the high reported rate of suicidal ideation and depression, mental health centers should provide additional training to clinicians and counselors on how to care for, screen, and manage suicidality in the MSM population. Additionally, knowledge of the psychological characteristics of MSM and development of consulting skills in clinic staff and Centers for Disease Control and Prevention (CDCs) related to working with MSM is needed to address their mental health needs, prevent suicidal behavior, and prevent HIV/AIDS infection and transmission. More generally, we should call on society to eliminate discrimination of the MSM population and provide more support and care to prevent suicidal ideation and attempts.

The study revealed that sexual victimization was associated with depressive symptoms among MSM, consistent with a study by Ratner et al.[28] Sexual violence from male partners and sexual abuse in childhood can have a negative effect on the mental health of MSM, which can increase the risk of HIV infection or other sexually transmitted infections. Consequently, health counselors and professionals should respond sensitively and give more care to victims of sexual victimization. For example, clinicians should identify the prevalence of sexual victimization among MSM, ask sensitive questions in the assessment of mental health, and deconstruct prejudicial beliefs about same-sex sexual violence.

Drinking alcohol was reported by forty percent and smoking by two thirds of our sample. Both drinking alcohol and smoking history were not associated with depressive symptoms. However, research indicates a strong association between mental health issues

For peer review only

and substance use disorders among the general population in studies of other countries.[59-60] Our study only indicated that being once drunk were associated with depressive symptoms among MSM. Further research is needed to verify the association between drinking alcohol, smoking, and depressive symptoms among Chinese MSM. Given there were few drug users in our sample and results of different studies are divergent, the finding that there was no association between drug use and present depressive symptoms in our study should be cautiously interpreted.

Previous studies have shown that high levels of sexual stigma can lead to internalized homophobia, and poor social support, all of which are risk factors for mental health conditions such as depression, anxiety, substance disorders, and suicidal ideation.[61-63,64] Men who have sex with men face social stigma in many settings, which can increase risk for HIV by limiting access to care.[65,66]. Thus, developing anti-stigma interventions and providing support that reduce homophobia and biphobia and encourage LGBT population rights, thereby reducing the amount of stigma, marginalization, and discrimination that MSM face in the first place would be very important in the future public health programmes. It is essential to change gradually social attitudes and reduce stigma and discrimination towards MSM populations. Health providers and MSM community organizations are valuable in providing social and emotional support to reduce stigma, alleviate mental disorder, and promote healthy behaviors. Besides, family members and friends from outside the MSM community also offer social resources after MSM disclose their sexual identity. Media campaigns against homophobia may

For peer review only - http://bmjopen.bmj.com/site/about/guidelines.xhtml

encourage broader acceptance of LGBT issues by portraying MSM in a non-discriminatory and normal manner.

There are some limitations to our study. First, these data were collected via self-report, and MSM might have concerns about their privacy and underreport some sensitive information in the rather homophobic and hostile environment of China. As such, this method may have resulted in an underestimation of the true prevalence of sensitive behaviors, such as unprotected sex, drug abuse, and sexual violence. Second, the causality of depressive symptoms and associated factors is not inferred due to the cross-sectional design, and the relevant information is subjected to recall bias, which may have an influence on the magnitude of associations with depression. Third, some important psychosocial factors, such as self-esteem, social support, and negative social relationships that might mediate or moderate the associations between depressive symptoms and related factors were not included in the research design. Additionally, stigma related to HIV and sexual stigma are important factors associated with depression[25] and were not examined. Fourth, different measures of depressive symptoms, such as the Self-Rating Depression Scale (SDS) and Beck Depression Inventory (BDI) may have provided results that differ from our study. Fifth, very few respondents with known HIV-positive status might report not knowing about their status before completing the study questionnaires for the incentive, which had an impact on the study result to some extent because depressive symptoms are very high and common among diagnosed HIV-infected people.[67-68] Thus, longitudinal research with a larger sample size,

For peer review only - http://bmjopen.bmj.com/site/about/guidelines.xhtml

employing more in-depth and complex measures of psychosocial and behavioral variables, and including such psychosocial factors as social support and social relationships, are needed to confirm our findings.

In response to our findings, mental health and substance abuse screening and counseling should be integrated into HIV prevention and treatment programs for the MSM population in China. Mental health issues are generally not given enough emphasis in the training of HIV/AIDS counselors in China, and we have had to provide supplementary training to staff from clinics and CDCs. More comprehensive intervention strategies that combine psychological, behavioral, and social aspects are needed to address the existing mental health issues, with special attention paid to suicidality and high-risk behaviors among MSM.

For peer review only

Author affiliations

[1]Center for Disease Control and Prevention of Zhejiang Province, Hangzhou, Zhejiang, China

[2]Department of Epidemiology & Biostatistics, School of Medicine, Ningbo University, Ningbo, Zhejiang, China

[3]Wenzhou Center for Disease Control and Prevention, Wenzhou, Zhejiang, China

[4] Lucheng Center for Disease Control and Prevention of, Wenzhou, Zhejiang, China.

Contributors SX, XP and QM were involved in the study design. HW, TJ, LH, SZ, DW, ZY, HZ and DZ performed the experiments. RL, XP and SX analysed the data. RL, XP and SX wrote the paper. XP, SX and QM critically revised the manuscript. All authors read and approved the final manuscript.

Acknowledgements We would like to thank Editage [www.editage.cn] for English language editing.

Funding Key Project on Social Development among S&T Major Project of Zhejiang Province, China (2013C03047-1) and General Program on Medicine and Health of Zhejiang Province, China (2016KYA066).

Competing interests None declared.

Patient consent Obtained.

Ethics approval The study was approved by the ethical review board of Zhejiang Provincial Center for Disease Control and Prevention.

Data sharing statement No additional data are available.

References

1. Peirce RS, Frone MR, Russell M, *et al*. A longitudinal model of social contact, social support, depression, and alcohol use. ***Health Psychol*** 2000;19:28-38.
2. Anderson AM, Ross MW, Nyoni JE, *et al*. High prevalence of stigma-related abuse among a sample of men who have sex with men in Tanzania: implications for HIV prevention. ***AIDS Care*** 2015;27:63-70.
3. Risher K, Adams D, Sithole B, *et al*. Sexual stigma and discrimination as barriers to seeking appropriate healthcare among men who have sex with men in Swaziland. ***J Int AIDS Soc*** 2013;16:18715.
4. Cochran SD, Bandiera FC, Mays VM. Sexual orientation-related differences in tobacco use and secondhand smoke exposure among US adults aged 20 to 59 years: 2003-2010 National Health and Nutrition Examination Surveys. ***Am J Public Health*** 2013;103:1837-44.
5. Cochran SD, Mays VM. Lifetime prevalence of suicide symptoms and affective disorders among men reporting same-sex sexual partners: results from NHANES III. ***Am J Public Health*** 2000;90:573-8.
6. Farmer GW, Bucholz KK, Flick LH, *et al*. CVD risk among men participating in the National Health and Nutrition Examination Survey (NHANES) from 2001 to 2010: differences by sexual minority status. ***J Epidemiol Community Health*** 2013;67:772-8.
7. Guerim LD, de Carvalho HW, Lara DR. The relationship between temperament and sexual orientation. ***J Affect Disord*** 2015;175:379-84.
8. Mimiaga MJ, O'Cleirigh C, Biello KB, *et al*. The effect of psychosocial syndemic production on 4-year HIV incidence and risk behavior in a large cohort of sexually active men who have sex with men. ***J Acquir Immune Defic Syndr*** 2015;68:329-36.
9. Tucker A, Liht J, de Swardt G, *et al*. An exploration into the role of depression and self-efficacy on township men who have sex with men's ability to engage in safer sexual practices. ***AIDS Care*** 2013;25:1227-35.
10. Grov C, Rendina HJ, Ventuneac A, *et al*. HIV risk in group sexual encounters: an event-level analysis from a national online survey of MSM in the U.S. ***J Sex Med*** 2013;10:2285-94.

For peer review only - http://bmjopen.bmj.com/site/about/guidelines.xhtml

11. Baggaley RF, White RG, Boily MC. HIV transmission risk through anal intercourse: systematic review, meta-analysis and implications for HIV prevention. ***Int J Epidemiol*** 2010;39:1048-63.

12. Jin F, Jansson J, Law M, *et al*. Per-contact probability of HIV transmission in homosexual men in Sydney in the era of HAART. ***AIDS*** 2010;24:907-13.

13. Ministry of Health, People's Republic of China. 2012 China AIDS Response Progress Report. Beijing: Ministry of Health; 2012. http://unaids.org.cn/pics/20120614140133.pdf

14. Ma Q, Zeng S, Xia S, *et al*. Risky sexual networks and concentrated HIV epidemics among men who have sex with men in Wenzhou, China: a respondent-driven sampling study. ***BMC Public Health*** 2015;15:1246.

15. Mills TC, Paul J, Stall R, *et al*. Distress and depression in men who have sex with men: the Urban Men's Health Study. ***Am J Psychiatry*** 2004;161:278-85.

16. Liu J, Gao YH, Liang ZM, *et al*. Depressive symptoms and associated sexual behaviors among men who have sex with men in Foshan, Guangdong province [in Chinese]. ***Zhonghua Liu Xing Bing Xue Za Zhi*** 2012;33:483-7.

17. Wang Y, Zhang HB, Xu J, Zhang GG, Yang HW, Fan J. Survey of depression status among men having sex with men[in Chinese]. ***J Prev Med Inform*** 2010;26:597-600.

18. Feng Y, Wu Z, Detels R, *et al*. HIV/STD prevalence among men who have sex with men in Chengdu, China and associated risk factors for HIV infection. ***J Acquir Immune Defic Syndr*** 2010;53 Suppl 1:S74-80.

19. Zheng YJ, Xu J, Zhang HB. Anxiety, depression and high-risk behaviors among men who have sex with men [in Chinese]. ***Chinese Mental Health Journal*** 2005;19:699-701.

20. Wu J, Lu CY, Hao YT, *et al*. Psychosocial status of MSM population in Guangzhou city [in Chinese]. ***Chin J Public Health*** 2011;27:476-8.

21. Fang F, Zhang SP, Wang ZW, *et al*. Epidemiological survey of depression among residents in a community of Shanghai [in Chinese]. ***Chin J Behav Med & Brain Sci*** 2009;18:740-2.

22. Graham LF, Braithwaite K, Spikes P, et al. Exploring the mental health of black men who have sex with men. ***Community Ment Health J*** 2009;45:272-84.

23. De Santis JP, Colin JM, Provencio Vasquez E, *et al*. The relationship of depressive symptoms, self-esteem, and sexual behaviors in a predominantly Hispanic sample of men who have sex with men. ***Am J Mens Health*** 2008;2:314-21.

24. Salomon EA, Mimiaga MJ, Husnik MJ, *et al*. Depressive symptoms, utilization of mental health care, substance use and sexual risk among young men who have sex with men in EXPLORE: implications for age-specific interventions. ***AIDS Behav*** 2009;13:811-21.

25. Secor AM, Wahome E, Micheni M, *et al*. Depression, substance abuse and stigma among men who have sex with men in coastal Kenya. ***AIDS*** 2015;29 Suppl 3:S251-9.

26. Hirshfield S, Wolitski RJ, Chiasson MA, et al. Screening for depressive symptoms in an online sample of men who have sex with men. ***AIDS Care*** 2008;20:904-10.

27. Semple SJ, Stockman JK, Goodman-Meza D, *et al*. Correlates of Sexual Violence Among Men Who Have Sex With Men in Tijuana, Mexico. ***Arch Sex Behav*** 2016. [Epub ahead of print]

28. Strathdee SA, Hogg RS, Martindale SL, et al. Determinants of sexual risk-taking among young HIV-negative gay and bisexual men. J Acquir Immune Defic Syndr Hum Retrovirol 1998;19:61-6.

For peer review only - http://bmjopen.bmj.com/site/about/guidelines.xhtml

29. Reisner SL, Mimiaga MJ, Bland S, *et al*. Problematic alcohol use and HIV risk among Black men who have sex with men in Massachusetts. ***AIDS Care*** 2010;22:577-87.

30. Hernandez MT, Sanchez MA, Ayala L, *et al*. Methamphetamine and cocaine use among Mexican migrants in California: the California-Mexico Epidemiological Surveillance Pilot. ***AIDS Educ Prev*** 2009;21:34-44.

31. Blashill AJ, O'Cleirigh C, Mayer KH, *et al*. Body mass index, depression and sexual transmission risk behaviors among HIV-positive MSM. ***AIDS Behav*** 2012;16:2251-6.

32. Houston E, Sandfort T, Dolezal C, *et al*. Depressive symptoms among MSM who engage in bareback sex: does mood matter? ***AIDS Behav*** 2012;16:2209-2215.

33. Liu JX, Choi K. Experiences of social discrimination among men who have sex with men in Shanghai, China. ***AIDS Behav*** 2006; 10(4 Suppl):S25–33. [PubMed: 16715344]

34. Zhang B, Chu Q. MSM and HIV/AIDS in China. ***Cell Research*** 2005;15:858–64.

35. Feng Y, Wu Z, Detels R. Evolution of men who have sex with men community and experienced stigma among men who have sex with men in Chengdu, China. ***J Acquir Immune Defic Syndr*** 2010; 53(Suppl 1):S98–S103.

36. Steward WT, Miege P, Choi K. Charting a moral life: the influence of stigma and filial duties on marital decisions among Chinese men who have sex with men. ***PLoS One*** 2013; 8:e71778.

37. Zang C, Guida J, Sun Y, *et al*. Collectivism culture, HIV stigma and social network support in Anhui, China: a path analytic model. ***AIDS Patient Care STDS*** 2014;28(8):452–58.

38. King M, Semlyen J, Tai SS, *et al*. A systematic review of mental disorder, suicide, and deliberate self harm in lesbian, gay and bisexual people. ***Bmc Psychiatry*** 2008;8:70.

39. Paul JP, Catania J, Pollack L, Stall R. Understanding childhood sexual abuse as a predictor of sexual risk-taking among men who have sex with men: the Urban Men's Health Study. ***Child Abuse Negl*** 2001;25:557–84.

40. Ratner PA, Johnson JL, Shoveller JA, *et al*. Non-consensual sex experienced by men who have sex with men: prevalence and association with mental health. ***Patient Educ Couns*** 2003;49:67-74.

41. Deuba K, Ekström AM, Shrestha R, et al. Psychosocial health problems associated with increased HIV risk behavior among men who have sex with men in Nepal: a cross-sectional survey. PLoS One 2013;8:e58099.

42. O'Carroll PW, Berman AL, Maris RW, *et al*. Beyond the Tower of Babel: a nomenclature for suicidology. ***Suicide Life Threat Behav*** 1996; 26: 237–52.

43. Friedman MR, Stall R, Silvestre AJ, *et al*. Effects of syndemics on HIV viral load and medication adherence in the multicentre AIDS cohort study. ***AIDS*** 2015;29:1087-96.

44. Zhang J, Wu Z, Fang G, *et al*. Development of the Chinese age norms of CES-D in urban area [in Chinese]. ***Chinese Mental Health Journal*** 2010;24:139-43.

45. Zhang Y, Jia C, Fang Z, *et al*. Study on depression symptom and reliability and validity of CES - D used in rural residents in Shandong province [in Chinese]. ***Chin J Public Health*** 2008;24:473-5.

46. Heckathorn DD. Respondent-driven sampling: a new approach to the study of hidden populations. ***Soc Probl*** 1997;44:174–99.

47. Volz E, Heckathorn DD. Probability based estimation theory for respondent driven sampling. ***Journal of Official Statistics***. 2008;24:79-97.

For peer review only - http://bmjopen.bmj.com/site/about/guidelines.xhtml

48. Wejnert C. An Empirical Test of Respondent-Driven Sampling: Point Estimates, Variance, Degree Measures, and Out-of-Equilibrium Data. ***Sociological Methodology*** 2009;39:73-116.

49. Sandfort TG, Bakker F, Schellevis FG, *et al*. Sexual orientation and mental and physical health status: findings from a Dutch population survey. ***Am J Public Health*** 2006;96:1119-25.

50. Stoloff K, Joska JA, Feast D, *et al*. A description of common mental disorders in men who have sex with men (MSM) referred for assessment and intervention at an MSM clinic in Cape Town, South Africa. ***AIDS Behav*** 2013;17 Suppl 1:S77-81.

51. Tucker A, Liht J, de Swardt G, *et al*. Homophobic stigma, depression, self-efficacy and unprotected anal intercourse for peri-urban township men who have sex with men in Cape Town, South Africa: a cross-sectional association model. ***AIDS Care*** 2014;26:882-9.

52. Shiu CS, Chen YC, Tseng PC, *et al*. Curvilinear relationship between depression and unprotected sexual behaviors among men who have sex with men. ***J Sex Med*** 2014;11:2466-73.

53. Golub SA, Starks TJ, Payton G, et al. The critical role of intimacy in the sexual risk behaviors of gay and bisexual men. ***AIDS Behav*** 2012;16:626-32.

54. Craft SM, Smith SA, Serovich JM, *et al*. Need fulfillment in the sexual relationships of HIV-infected men who have sex with men. ***AIDS Educ Prev*** 2005;17:217-26.

55. Li HM, Peng RR, Li J, *et al*. HIV incidence among men who have sex with men in China: a meta-analysis of published studies. ***PLoS One*** 2011;6:e23431.

56. Li R, Cai Y, Wang Y, *et al*. Psychosocial Syndemic Associated With Increased Suicidal Ideation Among Men Who Have Sex With Men in Shanghai, China. ***Health Psychol*** 2016;35:148-56.

57. Roura M, Urassa M, Busza J, *et al*. Scaling up stigma? The effects of antiretroviral roll-out on stigma and HIV testing. Early evidence from rural Tanzania. ***Sex Transm Infect*** 2009;85:308-12.

58. Sivasubramanian M, Mimiaga MJ, Mayer KH, *et al*. Suicidality, clinical depression, and anxiety disorders are highly prevalent in men who have sex with men in Mumbai, India: findings from a community-recruited sample. ***Psychol Health Med*** 2011;16:450-62.

59. Maslowsky J, Schulenberg JE, O'Malley PM, *et al*. Depressive symptoms, conduct problems, and risk for polysubstance use among adolescents: Results from US national surveys. ***Ment Health Subst Use*** 2013;7:157-69.

60. Schneider B, Kolves K, Blettner M, *et al*. Substance use disorders as risk factors for suicide in an Eastern and a Central European city (Tallinn and Frankfurt/Main). ***Psychiatry Res*** 2009;165:263-72.

61. Logie CH, Newman PA, Chakrapani V, *et al*. Adapting the minority stress model: associations between gender nonconformity stigma, HIV-related stigma and depression among men who have sex with men in South India. ***Soc Sci Med*** 2012;74:1261–8.,

62. Meyer IH. Prejudice, social stress, and mental health in lesbian, gay, and bisexual populations: conceptual issues and research evidence. ***Psychol Bull*** 2003;129:674–97.

63. Stahlman S, Grosso A, Ketende S, *et al*. Depression and Social Stigma Among MSM in Lesotho : Implications for HIV and Sexually Transmitted Infection Prevention. ***AIDS Behav*** 2015;19:1460–9.

64. Williamson IR. Internalized homophobia and health issues affecting lesbians and gay men. ***Health Educ Res*** 2000;15:97–107.

65. Stahlman S, Bechtold K, Sweitzer S, *et al*. Sexual identity stigma and social support among men

For peer review only - http://bmjopen.bmj.com/site/about/guidelines.xhtml

who have sex with men in Lesotho: a qualitative analysis. ***Reprod Health Matters*** 2015;23:127-35.
66. Zhao Y, Zhang L, Zhang H, *et al*. HIV Testing and Preventive Services Accessibility Among Men Who Have Sex With Men at High Risk of HIV Infection in Beijing, China. ***Medicine (Baltimore)*** 2015 ;94:e534.
67. Kanmogne GD, Qiu F, Ntone FE, *et al*. Depressive symptoms in HIV-infected and seronegative control subjects in Cameroon: Effect of age, education and gender. ***PLoS One***2017;12:e0171956.
68. Dalbó MJ, Manoel AL, Filho AO, *et al*. Depressive Symptoms and Associated Factors among People Living with HIV/AIDS. ***Journal of the International Association of Providers of Aids Care*** 2015;14:136-40.

Table 1. Sociodemographic characteristics and their associations with depressive symptoms among MSM in Wenzhou, China (N = 454)

Variables	Total n(%)	Major depressive symptoms n(%)	OR (95%CI)	*P* value
**Age(years)** (median = 33; IQR, 26–41)				
≤25	101(22.2)	39(38.6)	1.70(1.01-2.84)	0.044
26–35	168(37.0)	68(40.5)	1.84(1.17-2.87)	0.008
36 and older	185(40.7)	50(27.0)	1.00	
**Marital status**				
Never married	253(55.7)	97(38.3)	1.00	
Married/Divorced/Widowed	201(44.3)	60(29.9)	0.68(0.46-0.99)	0.049
**Education level**				
Junior school or lower	272(59.9)	87(32.0)	1.00	
Senior school	121(26.7)	50(41.3)	1.50(0.96-2.33)	0.074
College or higher	61(13.4)	20(32.8)	1.04(0.57-1.88)	0.904
**Monthly income (RMB)**				
<3000	194(42.7)	62(32.0)	1.00	

For peer review only - http://bmjopen.bmj.com/site/about/guidelines.xhtml

3000 and above	260(57.3)	95(36.5)	1.23(0.83-1.82)	0.310
**Duration of residence in Wenzhou (years)**				
≤3	220(48.6)	79(35.9)	1.00	
>3	233(51.4)	77(33.0)	0.88(0.60-1.30)	0.522
**Insurance**				
Insured	140(30.8)	54(38.6)	1.00	
Uninsured	314(69.2)	103(32.8)	0.78(0.51-1.18)	0.233
**Sexual orientation**				
Homosexual	184(40.5)	71(38.6)	1.34(0.91-1.99)	0.139
Heterosexual or bisexual or indeterminate	270(59.5)	86(31.9)	1.00	
**Places to seek for sexual partners**				
Venues	261(59.9)	85(32.6)	1.00	
Internet	175(40.1)	66(37.7)	1.25(0.84-1.87)	0.269
**Number of MSM which a participant knew in his circle of MSM friends locally** (median = 7; IQR, 3–15)				
>7	220(48.5)	74(33.6)	0.92(0.63-1.36)	0.681
≤7	234(51.5)	83(35.5)	1.00	

MSM = men who have sex with men, OR = odds ratio, CI = confidence interval, IQR = interquartile range.

Table 2. Sexual behavior and their associations with depressive symptoms among MSM in Wenzhou, China

(N = 454)

**Variables**	**Total n(%)**	**Major depressive symptoms n(%)**	**OR (95%CI)**	***P* value**

For peer review only

For peer review only - http://bmjopen.bmj.com/site/about/guidelines.xhtml

**Age of sexual debut with a man** (median = 24; IQR, 20–30)				
≤22	197(43.4)	70(35.5)	1.08(0.73-1.59)	0.709
>22	257(56.6)	87(33.9)	1.00	
**Anal intercourse with men in the past 6 months**				
Yes	386(85.0)	139(36.0)	1.56(0.88-2.79)	0.129
No	68(15.0)	18(26.5)	1.00	
**Number of anal intercourse male partners in the past 6 months** (median = 2; IQR, 1–3)				
≤2	307(67.6)	98(31.9)	1.00	
>2	147(32.4)	59(40.1)	1.43(0.95-2.15)	0.086
**Consistent condom use during anal sex in the past 6 months**				
Yes	174(45.1)	46(26.4)	1.00	
No	212(54.9)	93(43.9)	2.18(1.41-3.35)	<0.001
**Oral sex with men in the past 6 months**				
Yes	348(76.7)	124(35.6)	1.23(0.77-1.95)	0.394
No	106(23.3)	33(31.1)	1.00	
**Number of oral male sexual partners in the past 6 months**				
≤2	309(68.1)	97(31.4)	1.00	
>2	145(31.9)	60(41.4)	1.54(1.03-2.32)	0.038
**Consistent condom use during oral sex in the past 6 months**				
Yes	69(19.8)	24(34.8)	1.00	
No	279(80.2)	100(35.8)	1.05(0.60-1.82)	0.869
**Sex with women in the past**				
Yes	348(76.7)	115(33.0)	0.75(0.48-1.18)	0.213
No	106(23.3)	42(39.6)	1.00	
**Age of sexual debut with a woman**				
≤22	209(60.1)	71(34.0)	1.11(0.70-1.76)	0.653

For peer review only

>22	139(39.9)	44(31.7)	1.00	
**Sex with women in the past 6 months**				
Yes	167(36.8)	59(35.3)	1.05(0.71-1.57)	0.798
No	287(63.2)	98(34.1)	1.00	
**Number of female sex partners in the past 6 months**				
<2	407(90.0)	137(33.7)	1.00	
≥2	45(10.0)	19(42.2)	1.44(0.77-2.69)	0.254
**Consistent condom use during vaginal sex with women in the past 6 months**				
Yes	53(31.7)	21(39.6)	1.00	
No	114(68.3)	38(33.3)	0.76(0.39-1.50)	0.429

MSM = men who have sex with men, OR = odds ratio, CI = confidence interval, IQR = interquartile range.

Table 3. Substance use, psychosocial factors and their associations with depressive symptoms among MSM in Wenzhou, China (N = 454)

Variables	Total n(%)	Major Depressive symptoms n(%)	Odds Ratio (95%CI)	*P* value
**Smoking**				
Yes	284(62.6)	98(34.5)	0.99(0.67-1.48)	0.966
No	170(37.4)	59(34.7)	1.00	
**Drinking alcohol**				
Yes	188(41.4)	64(34.0)	0.96(0.65-1.42)	0.839
No	266(58.6)	93(35.0)	1.00	

For peer review only

**Being drunk once in the past year**				
Yes	87(46.3)	39(44.8)	2.47(1.33-4.59)	0.004
No	101(53.7)	25(24.8)	1.00	
**Wanting or needing to reduce drinking in the past year**				
Yes	48(55.2)	26(54.2)	2.36(0.99-5.67)	0.054
No	39(44.8)	13(33.3)	1.00	
**Drug use**				
Yes	17(3.7)	8(47.1)	1.72(0.65-4.55)	0.275
No	437(96.3)	149(34.1)	1.00	
**Suicidal ideation**				
Yes	73(16.1)	50(68.5)	5.57(3.24-9.57)	<0.001
No	381(83.9)	107(28.1)	1.00	
**Adult sexual violence from same-sex partners**				
Yes	36(7.9)	23(63.9)	3.75(1.84-7.63)	<0.001
No	418(92.1)	134(32.1)	1.00	
**Childhood sexual abuse**				
Yes	23(5.1)	14(60.9)	3.13(1.32-7.41)	0.009
No	431(94.9)	143(33.2)	1.00	

MSM = men who have sex with men, OR = odds ratio, CI = confidence interval.

Table 4. RDS-weighted multivariable analyses of factors associated with depressive symptoms among MSM in Wenzhou, China (N = 454)

Variable	AOR(95%CI)	*P* value	RDS-weighted AOR(95%CI)	*P* value
**Age(years)**				
≤25	1.26(0.62-2.54)	0.526	1.17(0.56-2.43)	0.681
26–35	1.74(1.01-3.01)	0.048	2.43(1.35-4.39)	0.003
36 and older	1		1	
**Marital status**				

For peer review only

For peer review only - http://bmjopen.bmj.com/site/about/guidelines.xhtml

Never married	1		1	
Married/Divorced/Widowed	0.73(0.43-1.24)	0.246	0.64(0.36-1.12)	0.119
**Education level**				
Junior school or lower	1		1	
Senior school	1.55(0.92-2.62)	0.103	2.01(1.16-3.49)	0.014
College or higher	1.08(0.53-2.20)	0.838	0.92(0.45-1.88)	0.825
**Monthly income (RMB)**				
<3000	1		1	
3000 and above	0.92(0.58-1.46)	0.714	0.65(0.40-1.03)	0.068
**Sexual orientation**				
Homosexual	1.05(0.67-1.66)	0.823	0.85(0.52-1.37)	0.499
Heterosexual or bisexual or indeterminate	1		1	
**Consistent condom use during anal sex with men in the past 6 with months**				
Yes	1		1	
No	2.13(1.32-3.45)	0.002	3.62(2.15-6.13)	<0.001
**Number of oral male sexual partners in the past 6 months**				
≤2	1		1	
>2	1.44(0.91-2.27)	0.12	1.75(1.09-2.80)	0.02
**Being drunk once in the past year**				
Yes	2.07(1.00-4.26)	0.049	2.89(1.36-6.14)	0.006
No	1		1	
**Suicidal ideation**				
No	1		1	
Yes	4.54(2.50-8.26)	<0.001	5.09(2.59-9.97)	<0.001
**Adult sexual violence from male sexual partners**				
No	1		1	
Yes	2.25(1.00-5.07)	0.051	2.57(1.03-6.73)	0.031
**Childhood sexual abuse**				
Yes	1.84(0.63-5.36)	0.263	1.89(0.34-10.57)	0.467
No	1		1	

MSM = men who have sex with men, AOR = adjusted odds ratio, CI = confidence interval; RDS=respondent-driving sampling.

For peer review only

For peer review only - http://bmjopen.bmj.com/site/about/guidelines.xhtml

STROBE 2007 (v4) Statement—Checklist of items that should be included in reports of *cross-sectional studies*

Section/Topic	Item #	Recommendation	Reported on page #
**Title and abstract**	1	(*a*) Indicate the study's design with a commonly used term in the title or the abstract	1-3
		(*b*) Provide in the abstract an informative and balanced summary of what was done and what was found	2-3
**Introduction**			
Background/rationale	2	Explain the scientific background and rationale for the investigation being reported	4-6
Objectives	3	State specific objectives, including any prespecified hypotheses	5-6
**Methods**			
Study design	4	Present key elements of study design early in the paper	6-7
Setting	5	Describe the setting, locations, and relevant dates, including periods of recruitment, exposure, follow-up, and data collection	6-7
Participants	6	(*a*) Give the eligibility criteria, and the sources and methods of selection of participants	6
Variables	7	Clearly define all outcomes, exposures, predictors, potential confounders, and effect modifiers. Give diagnostic criteria, if applicable	7-9
Data sources/ measurement	8*	For each variable of interest, give sources of data and details of methods of assessment (measurement). Describe comparability of assessment methods if there is more than one group	7-9
Bias	9	Describe any efforts to address potential sources of bias	*10*
Study size	10	Explain how the study size was arrived at	7
Quantitative variables	11	Explain how quantitative variables were handled in the analyses. If applicable, describe which groupings were chosen and why	10
Statistical methods	12	(*a*) Describe all statistical methods, including those used to control for confounding	10
		(*b*) Describe any methods used to examine subgroups and interactions	
		(*c*) Explain how missing data were addressed	
		(*d*) If applicable, describe analytical methods taking account of sampling strategy	10
		(*e*) Describe any sensitivity analyses	
**Results**			

For peer review only

Participants	13*	(a) Report numbers of individuals at each stage of study—eg numbers potentially eligible, examined for eligibility, confirmed eligible, included in the study, completing follow-up, and analysed	11-13
		(b) Give reasons for non-participation at each stage	
		(c) Consider use of a flow diagram	
Descriptive data	14*	(a) Give characteristics of study participants (eg demographic, clinical, social) and information on exposures and potential confounders	11-13
		(b) Indicate number of participants with missing data for each variable of interest	
Outcome data	15*	Report numbers of outcome events or summary measures	11-13
Main results	16	(*a*) Give unadjusted estimates and, if applicable, confounder-adjusted estimates and their precision (eg, 95% confidence interval). Make clear which confounders were adjusted for and why they were included	11-12
		(*b*) Report category boundaries when continuous variables were categorized	12-13
		(*c*) If relevant, consider translating estimates of relative risk into absolute risk for a meaningful time period	
Other analyses	17	Report other analyses done—eg analyses of subgroups and interactions, and sensitivity analyses	
**Discussion**			
Key results	18	Summarise key results with reference to study objectives	13
Limitations	19	Discuss limitations of the study, taking into account sources of potential bias or imprecision. Discuss both direction and magnitude of any potential bias	17
Interpretation	20	Give a cautious overall interpretation of results considering objectives, limitations, multiplicity of analyses, results from similar studies, and other relevant evidence	13-17
Generalisability	21	Discuss the generalisability (external validity) of the study results	17
**Other information**			
Funding	22	Give the source of funding and the role of the funders for the present study and, if applicable, for the original study on which the present article is based	18

*Give information separately for cases and controls in case-control studies and, if applicable, for exposed and unexposed groups in cohort and cross-sectional studies.

Note: An Explanation and Elaboration article discusses each checklist item and gives methodological background and published examples of transparent reporting. The STROBE checklist is best used in conjunction with this article (freely available on the Web sites of PLoS Medicine at http://www.plosmedicine.org/, Annals of Internal Medicine at http://www.annals.org/, and Epidemiology at http://www.epidem.com/). Information on the STROBE Initiative is available at www.strobe-statement.org.

For peer review only
